# Effects of Plucking Standards and Processing Methods on the Quality Characteristics of Summer Tea Leaves

**DOI:** 10.1002/fsn3.72445

**Published:** 2026-10-04

**Authors:** Jinlong Luo, Siyu Liao, Fengjiao Ding, Yuqiao Dai, Zhongying Liu, Tuo Zhang, Ting Yang, Qin Li, Shimao Fang, Yan Li, Wanping Fang, Qiang Shen

**Affiliations:** ^1^ Guizhou Tea Research Institute, Guizhou Academy of Agricultural Sciences Guiyang Guizhou China; ^2^ Wuzhi‐Xieying Tea Co., Ltd Wuzhishan China; ^3^ College of Horticulture Nanjing Agricultural University Nanjing China

**Keywords:** metabolomics, plucking standards, processing methods, summer tea, synergism effects

## Abstract

To improve the utilization value of summer tea resources, fresh summer tea leaves with different plucking standards were processed into four tea types (green, yellow, white, and black teas) to elucidate the effects of plucking maturity and processing methods on tea quality. Sensory evaluation, physicochemical analysis, electronic tongue, and metabolomics were employed to comprehensively characterize tea quality attributes. The tea samples made from one bud and one leaf showed superior sensory quality, especially in aroma, freshness, and sweetness. Processing significantly shaped flavor profiles: black tea reduced bitterness, white and yellow tea enhanced sweetness and umami, and green tea exhibited stronger umami and astringency characteristics. White and black tea processing also promoted key metabolite accumulation. Based on TAV (taste activity value) and rOAV (relative odor activity value), 10 key taste compounds (e.g., Asp, Glu, Thea, EGCG) and 10 key volatile metabolites (e.g., geraniol and dihydro‐2‐methyl‐3(2H)‐furanone) were identified, providing insights into the chemical mechanisms underlying tea quality development. The differential accumulation of taste compounds influenced taste characteristics, while variations in volatile metabolitescontributed to aroma diversity induced by different processing methods.

## Graphical abstract

This graphical abstract illustrated the combined effects of plucking standards and processing methods on summer tea quality. Summer fresh leaves (one bud with one leaf and one bud with three leaves) were processed into green, yellow, white and black teas. Sensory omics and chemometric analyzes revealed that plucking standards primarily influence taste attributes, while processing methods predominantly shaped volatile profiles.

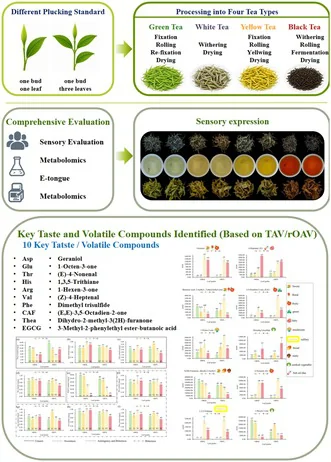

## 1 Introduction

China is one of the world's most important producers and consumers of tea (Su et al. 2022). Chinese abundant tea germplasm resources and diverse ecological conditions have given rise to a comprehensive tea ( *Camellia sinensis* (L.) O. Kuntze) classification system comprising six major tea types, including green tea, yellow tea, white tea, oolong tea, dark tea, and black tea. Tea quality is generally considered to be the result of the synergistic effects of multiple factors, including tea cultivar, ecological environment, cultivation practices, and processing technology (Fu et al. 2024). Beyond these factors, the season of fresh tea leaves production is also a crucial determinant of tea quality (Wang, Li, et al. 2021). Elevated summer temperatures promote the accumulation of bitter and astringent taste‐related compounds, such as tea polyphenols and caffeine, while reducing the levels of amino acids and aroma precursors associated with fresh and sweet flavors, resulting in teas characterized by limited aroma complexity and pronounced bitterness and astringency (Liang et al. 2023; Liu et al. 2025; Wei et al. 2022; Yang et al. 2013). These quality limitations directly constrain the enhancement of the market value and economic benefits of summer tea. Improving processing strategies represents effective approaches to overcoming the inherent quality constraints of summer tea and achieving its efficient utilization (Sun et al. 2024; Wang, Li, et al. 2021).

The intrinsic quality of fresh tea leaves, determined by factors such as tenderness, harvesting time, and bud–leaf composition, fundamentally determines their initial physicochemical characteristics. These attributes establish the foundation for downstream processing and the sensory and chemical quality formation of finished teas (Chen et al. 2024). Processing methods are critical in transforming fresh tea leaves into consumable tea products. Tea processing consists of a series of complex yet controllable enzymatic and non‐enzymatic reactions, encompassing key operations such as withering, bruising (rolling or shaking), fixation, yellowing, fermentation, pile fermentation, and drying (Fang et al. 2022; Guo et al. 2021; Lin et al. 2021; Ren et al. 2024; Shi et al. 2023; Wu et al. 2020). Through these physicochemical reactions, quality‐related constituents in fresh tea leaves undergo extensive transformation, enabling the targeted regulation of flavor profiles. Ultimately, these processes establish the material basis for the characteristic sensory attributes of specific tea types, endowing them with distinctive color, aroma, taste, and appearance (Hu et al. 2022). Accordingly, plucking and processing represent two closely coupled stages, which jointly affect the information of summer tea flavor. Only when appropriate plucking strategies are matched with compatible processing methods can the full potential of fresh tea leaves be maximized. Conversely, the design and optimization of tea processing routes should explicitly take into account the intrinsic characteristics of different fresh leaf materials (Feng et al. 2024; Wen, Sun, Zhang, et al. 2023). Nevertheless, comprehensive and systematic analyzes of the mutual influence between plucking standards and processing techniques in summer fresh tea leaves remain limited. Especially, the matching relationships between fresh leaf quality attributes and processing routes for multi‐type tea production using summer fresh leaves are still poorly understood. Consequently, tea manufacturing practices remain predominantly reliant largely on empirical knowledge, which hampers the implementation of precision, standardization, and value‐added processing strategies for summer fresh tea leaves (Huang et al. 2024).

Quantitative determination of physicochemical quality‐related compounds and descriptive sensory evaluation have long been regarded as conventional approaches for assessing tea quality (Fan et al. 2021). In recent years, omics‐based analyzes have increasingly been applied to elucidate the material basis of tea quality attributes and the underlying mechanisms of quality formation, thereby overcoming the limitations of conventional methods, such as strong subjectivity and restricted mechanistic interpretation (Luo et al. 2025). The integration of descriptive sensory analysis with metabolomics, including both volatile and non‐volatile metabolomic profiling, provides a systematic and objective approach for elucidating tea quality and its formation mechanisms. This combined strategy enables the comprehensive screening and identification of key metabolites that contribute critically to tea flavor and, through multivariate statistical analyzes, reveals the intrinsic relationships between sensory attributes and metabolite composition (Qin et al. 2020; Wang, Cao, et al. 2021). Such an approach offers a robust experimental framework for comprehensively investigating the effects of plucking standards and processing methods on the quality of teas produced from summer fresh tea leaves.

To systematically investigate the mutual influence effect of fresh leaf harvesting standards and tea processing techniques, and to achieve the targeted transformation of summer tea fresh leaves into diversified tea products with typical flavor characteristics, this study aimed to improve the quality of summer tea and promote the high‐value utilization of summer tea fresh leaves. Summer tea fresh leaves with different harvesting standards, including one bud with one leaf and one bud with two to three leaves, were selected as raw materials and processed into green tea, white tea, yellow tea, and black tea following their respective classical processing procedures. Widely targeted metabolomics was employed to comprehensively characterize the profiles of volatile and non‐volatile metabolites in all tea samples. An electronic tongue system was used to objectively evaluate the overall taste profiles of the tea infusions. Taste value analysis (TVA) and relative odor activity value (rOAV) analyzes were applied to identify key differential taste‐active and aroma‐active compounds among teas produced from different harvesting–processing combinations of harvesting standards and processing methods. Furthermore, multivariate statistical analysis was conducted to establish correlations between key quality‐related compounds and core sensory attributes, thereby elucidating the mechanisms underlying the combined regulation of summer tea quality by fresh leaf harvesting standards and processing techniques. Overall, this study provides systematic insights into the combined effects of harvesting standards and processing methods on tea quality and offers a theoretical basis for the quality improvement and high‐value utilization of summer tea fresh leaves.

## 2 Materials and Methods

### 2.1 Chemicals and Materials

The summer tea fresh leaves used in this study were collected on June 15, 2025, from Nayong County Tea Industry Co. Ltd., located in Bijie City, Guizhou Province, China. The tea cultivar was “Fudingdabaicha” ( *Camellia sinensis* (L.) O. Kuntze). The fresh leaves were collected according to two plucking standards: one bud with one leaf and one bud with two to three leaves. Catechin and caffeine standards were purchased from Cato Research Chemicals (USA), and amino acid standards were obtained from Waters Corporation (USA). Chromatographic‐grade acetic acid, acetonitrile, and methanol were purchased from Tedia Company (USA). Ethyl decanoate was obtained from Sigma‐Aldrich (USA).

### 2.2 Tea Sample Preparation

Tea sample was manufactured at Mingsheng Tea Industry Co. Ltd., located in Nayong County, Bijie City, Guizhou Province, China. Fresh leaves were processed according to the procedures corresponding to conventional manufacturing procedures for green tea, yellow tea, white tea, and black tea, respectively (Figure 1).

**FIGURE 1:**
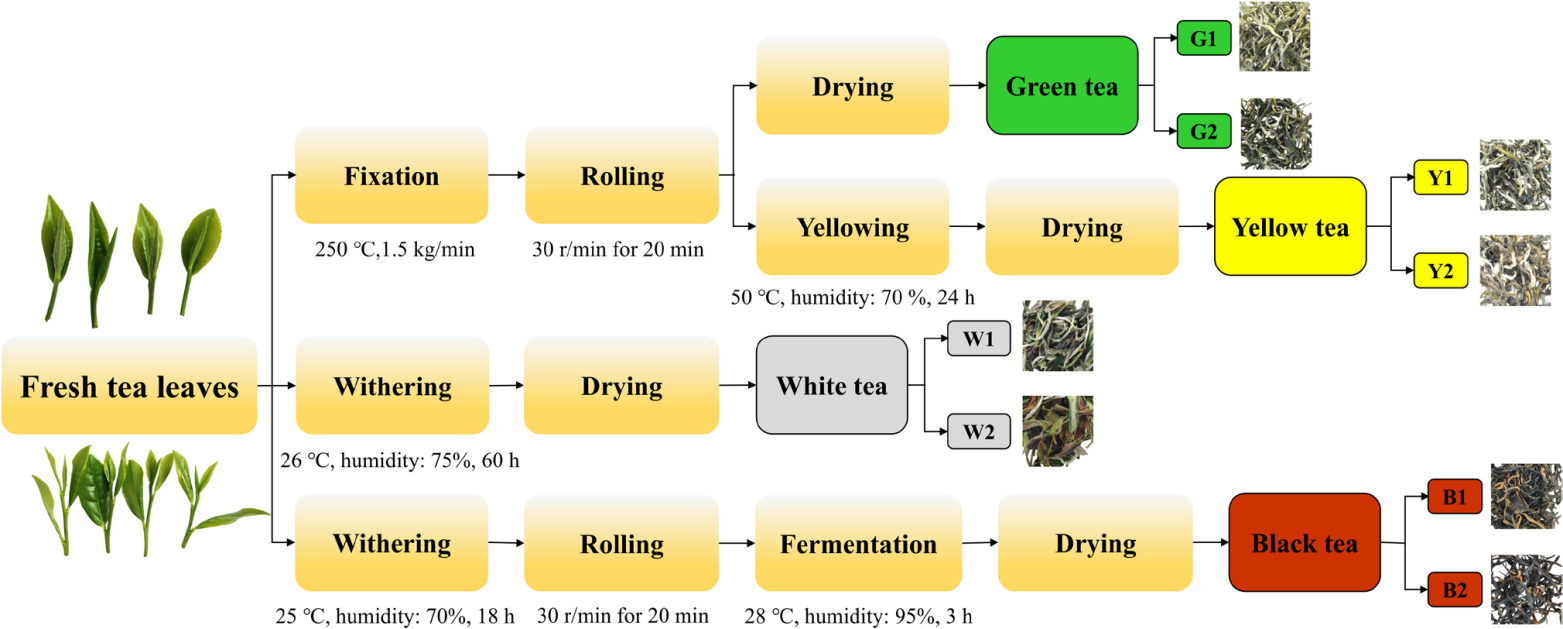
The schematic illustration of the processing procedures for four types of summer tea. G1 and G2 represented green tea produced from summer fresh tea leaves with one bud and one leaf, and on one bud with two to three leaves, respectively. Y1 and Y2 represented yellow tea produced from summer fresh tea leaves with one bud and one leaf, and on one bud with two to three leaves, respectively. W1 and W2 represented white tea produced from summer fresh tea leaves with one bud and one leaf, and on one bud with two to three leaves, respectively. B1 and B2 represented black tea produced from summer fresh tea leaves with one bud and one leaf, and on one bud with two to three leaves, respectively.

The manufacture for green tea was as follows: fresh plucked leaves (FPTs), fixation (250°C, feeding rate 1.5 kg/min), cooling (25°C, 30 min), rolling (at 30 r/min for 20 min), re‐fixation (100°C, 40 min), re‐cooling (25°C, 2 h), and drying (80°C, drying until the water content was 5%). The manufacture for yellow tea was as follows: FPTs, fixation (250°C, feeding rate 1.5 kg/min), cooling (25°C, 30 min), rolling (at 30 r/min for 20 min), re‐fixation (100°C, 40 min), yellowing (40°C, humidity: 50%, 12 h), pre‐drying (100°C, 40 min), re‐yellowing (50°C, humidity: 70%, 24 h), and drying (80°C until the final moisture content reached 5%). The manufacture for white tea was as follows: FPTs, withering (sunlight withering for 40 min, followed by indoor natural withering for 60 h at a relative humidity of 75% and a temperature of 26°C), and drying (80°C, drying until the water content was 5%). The manufacture for black tea was as follows: FPTs, withering (25°C, humidity: 70%, 18 h), rolling (at 30 r/min for 60 min), fermentation (28°C, humidity: 95%, 3 h), and drying (initial drying for 10 min at 120°C, cooling for 20 min at room temperature, re‐drying for 120 min at 80°C until the water content was 5%).

Green tea, yellow tea, white tea, and black tea prepared from fresh leaves with one bud and one leaf were designated as G1, Y1, W1, and B1, respectively. Similarly, teas were produced from fresh leaves with one bud and two to three leaves were designated as G2, Y2, W2, and B2, respectively. Three replications were considered. All samples were stored at −80°C for subsequent analyzes.

### 2.3 Sensory Analysis

The sensory evaluation of these summer tea samples was conducted according to previously reported methods (Hua et al. 2024; Luo et al. 2025). Sensory evaluation was used to horizontally compare the effects of raw material tenderness and processing methods on the expression of sensory quality in tea samples using a unified method and scale. All tea samples were food‐grade. Appropriate measures were implemented to protect the rights and interests of all participants, including voluntary participation without coercion, full disclosure of the study requirements and potential risks, obtaining informed consent from all participants, and allowing participants to withdraw from the study at any time. The sensory judge panel consisted of 5 trained tea judges from the Guizhou Tea Research Institute who participated in the assessment. It involved two males and three females, who were aged 20~40 years. The sensory evaluation team was independent of this research team. The evaluations were performed in a clean, dry room maintained at 26°C ± 1°C.

Tea infusions were prepared following a standardized brewing procedure and presented to the panelists in randomized order with three‐digit codes. Each sample was evaluated in triplicate. The tea samples were attributed with the quality factors of tea, including appearance, infusion color, aroma, taste, and infused leaves. The total sensory evaluation scores of these tea samples in our research were calculated using weighted coefficients, with appearance (25%), infusion color (10%), aroma (25%), taste (30%), and infused tea leaf (10%). The total score for the tea sensory evaluation was scored on a 100‐point scale.

### 2.4 Determination of Quality‐Relative Compounds

The contents of individual catechin components [EGCG (epigallocatechin gallate), ECG (epicatechin gallate), EGC (epigallocatechin), EC (epicatechin), and C (catechin)], CAF (caffeine), and individual amino acid components [Thea (theanine), Asp (aspartic acid), Glu (glutamic acid), Ser (serine), Gly (glycine), Thr (threonine), Ala (alanine), Pro (proline), His (histidine), Arg (arginine), Tyr (tyrosine), Val (valine), Met (methionine), Ile (isoleucine), Leu (leucine), Phe (phenylalanine), Lys (lysine), Cys (cysteine), and HYP (hydroxyproline)] were determined according to the method reported by Yang et al. (2024).

The determination methods for the contents of individual catechin components and CAF were described below. Tea powder (0.2 g) was extracted twice with 5 mL of 70% (v/v) methanol preheated to 70°C for 10 min with intermittent shaking (every 5 min). After cooling to room temperature, the extract was centrifuged at 3500 rpm for 10 min, combined, and adjusted to a final volume of 10 mL. The solution was filtered through a 0.45 μm membrane. An aliquot (1 mL) of the extract was mixed with 2 mL of a stabilizing solution (EDTA‐2Na, 10 mg/mL; ascorbic acid, 10 mg/mL; acetonitrile; ultrapure water = 1:1:2:16, v/v), followed by filtration through a 0.45 μm membrane before HPLC analysis. Catechins [EGCG (epigallocatechin gallate), EC (epicatechin), EGC (epigallocatechin), and ECG (epicatechin gallate)] and caffeine were quantified using an LC‐2030C Plus HPLC system (Shimadzu, Japan) equipped with a Shim‐pack GIST C18 column (4.6 × 250 mm, 5 μm). The mobile phase consisted of solvent A (9% acetonitrile, 2% acetic acid, and 0.2% EDTA‐2Na solution, 10 mg/mL) and solvent B (80% acetonitrile, 2% acetic acid, and 0.2% EDTA‐2Na solution, 10 mg/mL). The column temperature was maintained at 35°C with a flow rate of 1.0 mL/min. The gradient elution was as follows: 0–10 min, 100% solvent A; 10–25 min, 100%–68% solvent A; 25–35 min, 68%–100% solvent A; 35–45 min, 100% solvent A. Detection was performed at 278 nm with an injection volume of 10 μL.

Amino acids were derivatized using the AccQ‐Fluor reagent kit according to the manufacturer's instructions. The derivatized samples were prepared according to the instructions provided with the reagent kit. 10 μL of the derivatized sample was injected into an LC‐2030C Plus HPLC system (Shimadzu, Japan) equipped with a fluorescence detector (FLD) for the quantification of 18 amino acids. Separation was achieved on an AccQ·Tag column (3.9 × 150 mm, Waters, Ireland) using solvent A (10% AccQ·Tag amino acid analysis concentrate), solvent B (acetonitrile), and solvent C (ultrapure water) at a column temperature of 32°C and a flow rate of 1.0 mL/min. The gradient elution program was as follows: 0 min, 100% A; 0–17 min, 100%–90% A; 17–24 min, 90%–80% A; 24–34 min, 80%–68% A; 34–37 min, 68%–0% A; 38–45 min, 0%–100% A, with corresponding changes in solvents B and C. Detection was performed at 278 nm. Total free amino acids (FAAs) were calculated as the sum of the 18 amino acids. Sweetness amino acids (SAAs) included Gly, Ala, Pro, Ser, Thr, and Lys; bitter amino acids (BAAs) included Arg, Ile, Val, Tyr, Phe, Leu, Met, and His; umami amino acids (UAAs) included Glu, Asp, and L‐theanine.

### 2.5 Taste Activity Value Analysis

Quality‐related compounds in tea contribute to its diverse taste attributes; catechins are strongly associated with bitterness, caffeine is closely related to briskness, and amino acids are primarily responsible for umami taste. Taste activity value (TAV) is defined as the ratio of the concentration of a taste‐active compound to its taste threshold in water and is widely used to evaluate the potential contribution of specific compounds to taste perception. A TAV < 1 indicates that the compound has little or no direct contribution to taste, whereas a TAV > 1 indicates a perceptible contribution, with higher values corresponding to a greater impact on overall taste intensity (Zhang et al. 2022). TAV is applied to quantify the contribution of individual taste compounds to the overall taste of a sample and is calculated using the following equation:

$$\mathrm{TAV}=\frac{Tx}{T}$$

where *Tx* represents the concentration of the taste‐active compound (mg/g), and *T* denotes the taste threshold of the compound (mg/g).

### 2.6 E‐Tongue Analysis

E‐tongue (electronic tongue) enables rapid, objective, and standardized discrimination of tea samples with different categories and quality levels by accurately identifying the profiles of non‐volatile flavor compounds in terms of both composition and abundance (Zhu, Jiang, et al. 2025). The E‐tongue analysis was performed according to the method described by Yang et al. (2024). E‐tongue analysis was conducted using SA‐402B electronic tongue (Insent, Japan). For E‐tongue analysis, tea infusions were prepared by steeping 3.0 g of tea powder in 150 mL of boiling water for 5 min, followed by filtration. Subsequently, 35 mL of the filtrate was transferred into a 50 mL dedicated cup for measurement, with data acquisition conducted alternately with sensor cleaning. Each tea sample was analyzed in four independent replicates. For each sample, the responses for bitterness, astringency, and umami were measured four times. In contrast, due to the longer response stability time of the sweetness sensor, an additional parallel measurement is added to ensure the accuracy of the data. The E‐tongue response values were calculated using the following equation:

V=Vs.−Vr

where V is the relative potential value of the sample solution, Vs. is the output value of the membrane potential signal of the taste sensor in the sample solution, and Vr is the output value of the membrane potential signal of the taste sensor in the reference solution.

### 2.7 Volatile Metabolomics Analysis

#### 2.7.1 HS‐SPME Extraction

Volatile metabolomics analysis was conducted using headspace solid‐phase microextraction coupled with gas chromatography–mass spectrometry (HS‐SPME–GC–MS) according to the method reported by Ding et al. (2025). The tea samples were ground under liquid nitrogen to obtain a homogeneous powder. A 500 mg aliquot of the powder was placed into a 20 mL headspace vial together with saturated NaCl solution and 10 μL of internal standard solution (3‐hexanone‐2,2,4,4‐d₄, 50 μg/mL; CAS: 24588‐54‐3). The vial was equilibrated at 60°C for 5 min, after which a 120 μm DVB/CAR/PDMS fiber (Agilent, USA) was exposed to the headspace at 60°C for 15 min. Before extraction, the fiber was conditioned at 250°C for 5 min.

#### 2.7.2 GC–MS Analysis

After extraction, the SPME fiber was thermally desorbed in the GC injection port at 250°C for 5 min. Volatile metabolites were analyzed using an Agilent 7890B gas chromatograph coupled with an Agilent 7000D mass spectrometer (Agilent, USA), equipped with a DB‐5MS capillary column (30 m × 0.25 mm i.d. × 0.25 μm film thickness; Agilent, USA). High purity helium (> 99.999%) was used as the carrier gas at a constant flow rate of 1.2 mL/min. The GC oven temperature was initially held at 40°C for 3.5 min, then increased to 100°C at 10°C/min, raised to 180°C at 7°C/min, and finally ramped to 280°C at 25°C/min, where it was maintained for 5 min. Mass spectrometric detection was performed in electron impact (EI) mode at 70 eV, with the quadrupole analyzer, ion source, and transfer line temperatures set at 150°C, 230°C, and 280°C, respectively.

#### 2.7.3 Identification and Quantification of Volatile Metabolites

Mass spectrometric data processing was performed using MassHunter software (version 12.0). Volatile metabolites were tentatively annotated by comparing their mass spectra with entries in the Metware database using MassHunter software (version 12.0). Features with unreliable signals or poor repeatability were removed during data processing. The annotated metabolites were considered putatively identified because authentic standard confirmation was not performed. Relative quantification was achieved using an internal standard–based calibration method, normalized to the peak areas of the internal standard, as previously described by Ding et al. (2025).

### 2.8 Relative Odor Activity Value Analysis

Relative odor activity value (rOAV) is used to screen key volatile metabolites in foods by relating their concentrations to their sensory thresholds, thereby evaluating the contribution of individual compounds to the overall aroma of tea samples. The rOAV method, as a semi‐quantitative preliminary screening strategy, was used to identify important volatile metabolites among different tea types (Ding et al. 2025). The rOAV was calculated according to the following equation, as reported previously (Ding et al. 2025; Huang et al. 2022):

$$\text{rOAV}=\frac{C}{T}$$

where rOAV denotes the relative odor activity value of a given volatile compound, *C* represents its relative concentration (μg/g), and *T* indicates the odor threshold of the compound in water (μg/g).

### 2.9 Data Analysis

Data processing and visualization were performed using Origin 2021, including the construction of mirror histograms. Statistical analysis was performed using one‐way analysis of variance (ANOVA) using SPSS 21 (IBM Corp., Armonk, NY, USA). Multivariate analyzes, including orthogonal partial least squares discriminant analysis (OPLS‐DA), ring plots, heatmap visualization, and Venn diagram analysis, were carried out using the Metware Cloud platform (accessed on 15 October 2025).

## 3 Results and Discussion

### 3.1 Sensory Differences Among Tea Processing Methods

Fresh leaf plucking standards and tea processing methods significantly influenced the sensory quality of teas produced from summer fresh leaves. Based on sensory evaluation (Table 1 and Figure 2), the overall sensory scores of tea samples produced under different plucking standards and processing methods decreased in the following order: G1 (93.57) > G2 (89.72), W1 (92.35) > W2 (86.74), Y1 (92.41) > Y2 (89.01), and B1 (93.48) > B2 (89.85). Tea samples prepared from summer tea leaves with one bud and one leaf plucking standard (G1, Y1, W1, and B1) generally exhibited superior quality compared with those produced from one bud with two to three leaves (G2, Y2, W2, and B2). These results indicated that leaf maturity at harvest significantly influences the final tea quality, with more tender raw leaves contributing to improved sensory performance.

**TABLE 1:** Sensory evaluation results of summer tea processed using different processing methods.

Tea sample	Appearance (25%)		Aroma (25%)		Liquor color (10%)		Taste (30%)		Infused leaves (10%)		Score
	Comments	Score	Comments	Score	Comments	Score	Comments	Score	Comments	Score	
G1	Tightly rolled and slender strands; Glossy surface; Tender green appearance; Visible trichomes	23.33 ± 0.14	Tender; Chestnut‐like; Floral	23.33 ± 0.14	Tender green; Bright	9.37 ± 0.06	Mellow; Briskness	28.20 ± 0.30	Bright; Tender yellow‐green; Soft texture	9.33 ± 0.06	93.57 ± 0.15
G2	Relatively tight and slender strands with slight rolling; Moderately glossy surface; Green appearance with some visible trichomes	22.17 ± 0.14	Chestnut‐like	22.42 ± 0.14	Green; Bright	9.17 ± 0.06	Mellow; Moderate briskness	27.1 ± 0.17	Bright; Yellow‐green; Moderate softness	8.87 ± 0.06	89.72 ± 0.19
Y1	Tightly rolled and slender strands; Glossy appearance with prominent tips; Light greenish‐yellow color	23.00 ± 0.25	Tender; Chestnut‐like; Sweet	23.04 ± 0.19	Tender yellow; Bright	9.27 ± 0.06	Mellow; Refreshing	27.8 ± 0.17	Bright; Tender yellow; Soft texture	9.30 ± 0.03	92.41 ± 0.64
Y2	Relatively tight and slender strands with slight rolling; Moderately glossy appearance; Brownish‐yellow color	21.75 ± 0.25	Chestnut‐like; Sweet	22.46 ± 0.19	Yellow; Bright	8.83 ± 0.06	Mellow; Relatively strong briskness	27.1 ± 0.17	Bright; Yellow; Moderate softness	8.87 ± 0.06	89.01 ± 0.52
W1	Natural floral‐branch shape; Prominent trichomes; Silvery‐green appearance	23.08 ± 0.14	Fresh and sweet; Pronounced pekoe aroma	22.46 ± 0.19	Light orange‐yellow; Bright	8.95 ± 0.05	Fresh; Mellow; Pronounced pekoe flavor	28.05 ± 0.15	Bright; Grayish green; Soft texture	8.93 ± 0.06	92.35 ± 0.35
W2	Natural floral‐branch shape; Visible trichomes; Grayish‐green color tinged with yellow‐brown	20.50 ± 0.25	Fresh; Pekoe aroma	21.96 ± 0.19	Orange‐yellow; Bright	8.78 ± 0.03	Sweet; Mellow; Relatively strong briskness	27.10 ± 0.17	Bright; Green; Moderate softness	8.40 ± 0.10	86.74 ± 0.18
B1	Tightly rolled and slender strands; Prominent golden trichomes; Glossy brownish appearance	23.46 ± 0.19	Tender; Sweet; Floral; Fruity	23.25 ± 0.13	Red; Bright	9.33 ± 0.06	Fresh; Sweet; and full‐bodied; Briskness	28.1 ± 0.17	Bright; Red; Soft texture	9.33 ± 0.06	93.48 ± 0.20
B2	Exhibited relatively tight and slender rolled strands; Golden trichomes; Moderately glossy brownish appearance	22.46 ± 0.19	Sweet	22.13 ± 0.13	Orange‐red; Bright	9.17 ± 0.06	Sweet; Briskness	27.1 ± 0.17	Bright; Red; Moderate softness	9.00 ± 0.09	89.85 ± 0.51

*Note:* G1 and G2 represented green tea produced from summer fresh tea leaves with one bud and one leaf and one bud with two to three leaves, respectively. Y1 and Y2 represented yellow tea produced from summer fresh tea leaves with one bud and one leaf and one bud with two to three leaves, respectively. W1 and W2 represented white tea produced from summer fresh tea leaves with one bud and one leaf and one bud with two to three leaves, respectively. B1 and B2 represented black tea produced from summer fresh tea leaves with one bud and one leaf and one bud with two to three leaves, respectively. The data were expressed as sensory evaluation scores in the form of “mean ± standard” deviation.

**FIGURE 2:**
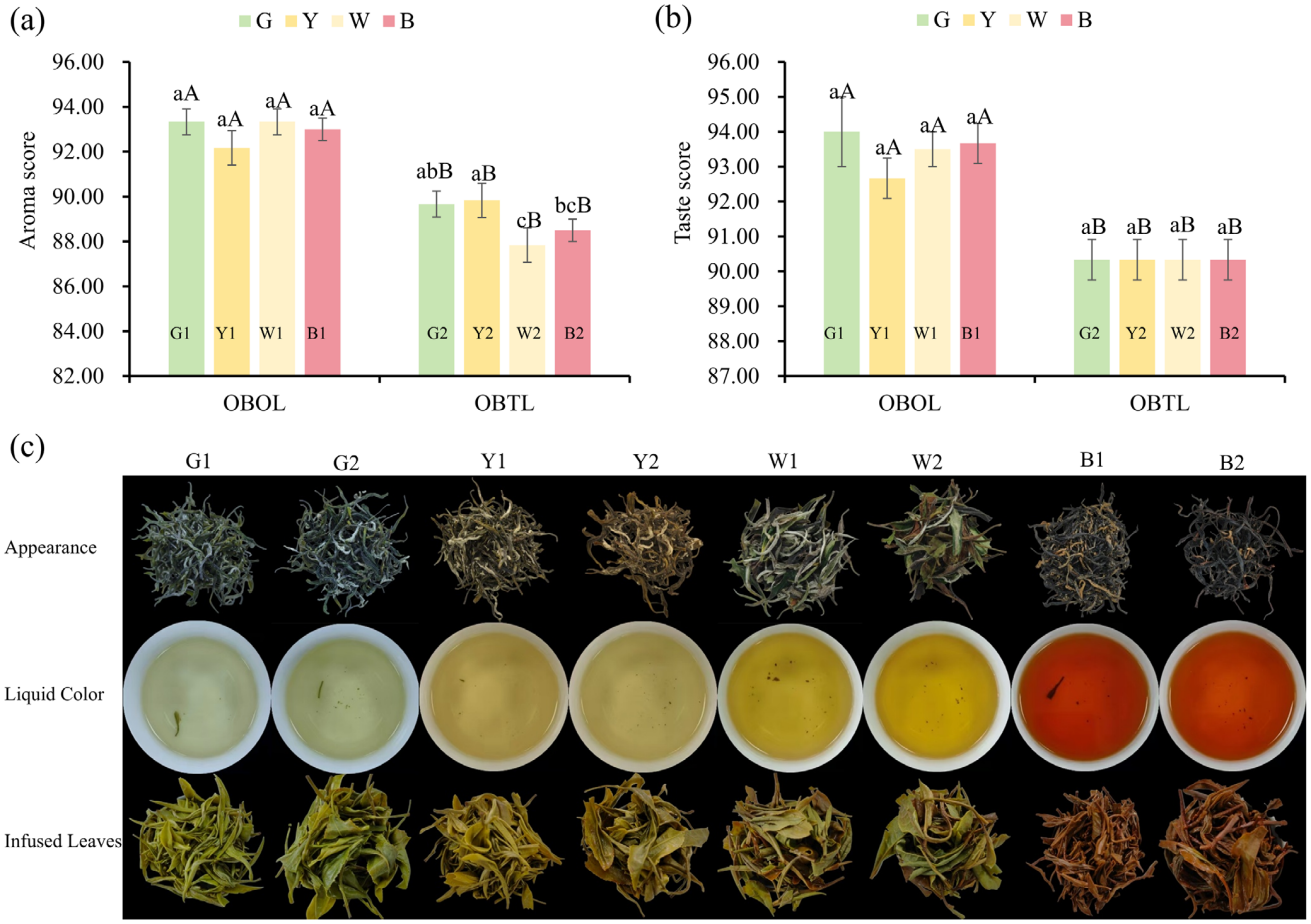
Sensory evaluation diagram of summer tea processed by four tea processing methods. (a) Aroma rating bar chart, (b) tasting rating bar chart, and (c) the appearance of tea liquor and infused leaves of samples treated by different drying temperatures and times. G1 and G2 represented green tea produced from summer fresh tea leaves with one bud and one leaf, and on one bud with two to three leaves, respectively. Y1 and Y2 represented yellow tea produced from summer fresh tea leaves with one bud and one leaf, and on one bud with two to three leaves, respectively. W1 and W2 represented white tea produced from summer fresh tea leaves with one bud and one leaf, and on one bud with two to three leaves, respectively. B1 and B2 represented black tea produced from summer fresh tea leaves with one bud and one leaf, and on one bud with two to three leaves, respectively. OBOL referred to fresh tea shoots with one bud and one leaf. OBTL referred to fresh tea shoots with one bud and two to three leaves. Lowercase letters denote significant differences in sensory quality among teas from the same fresh leaf grade but different processing methods (*p* < 0.05). Uppercase letters denote significant differences among teas from the same processing method but different fresh leaf grades (*p* < 0.05).

All tea samples exhibited the characteristic sensory attributes typical of their respective tea types (Table 1 and Figure 2) (Zhou et al. 2022). To further elucidate the effects of fresh leaf tenderness on tea quality, sensory performance was analyzed within individual tea types. Different tea types exhibited distinct responses to fresh leaf grades, with white tea being the most sensitive to leaf tenderness, followed by black and green teas, while yellow tea showed relatively stable sensory quality. For green tea (G1 and G2), a decrease in fresh leaf tenderness resulted in reduced aroma complexity and diminished briskness of taste. Specifically, the tender and floral aromas observed in G1 were not detected in G2. For yellow tea (Y1 and Y2), a decrease in fresh leaf tenderness primarily reduced the intensity of sweet aroma and taste, as well as the mellowness of the taste. In white tea (W1 and W2), reduced fresh leaf tenderness weakened the pekoe aroma and umami perception but enhanced sweetness. For black tea (B1 and B2), a decrease in fresh leaf tenderness resulted in changes in aroma profile and a reduction in umami perception. Specifically, B1 was characterized by a tender and sweet aroma with floral and fruity notes, whereas B2 mainly exhibited a sweet aroma.

A pronounced plucking–processing mutual influence effect was observed, as the identical raw material produced markedly different flavor profiles under different processing methods. These results indicate that appropriate processing can partially compensate for the quality limitations of lower‐grade summer fresh leaves. However, high‐tenderness fresh leaves remain the fundamental basis for producing high‐quality tea products. These findings highlight a potential processing compensation mechanism, whereby suitable processing strategies can partially alleviate the sensory disadvantages of less tender summer leaves by reshaping their aroma and taste profiles.

### 3.2 E‐Tongue Analysis

To objectively evaluate the effects of plucking standards and processing methods on tea taste quality, an electronic tongue was employed to analyze bitterness, astringency, umami, and sweetness (Figure 3 and Table S1). The results demonstrated that both plucking standards and processing methods exerted significant effects on tea taste quality. E‐tongue analysis objectively revealed a pronounced mutual influence between the two factors.

**FIGURE 3:**
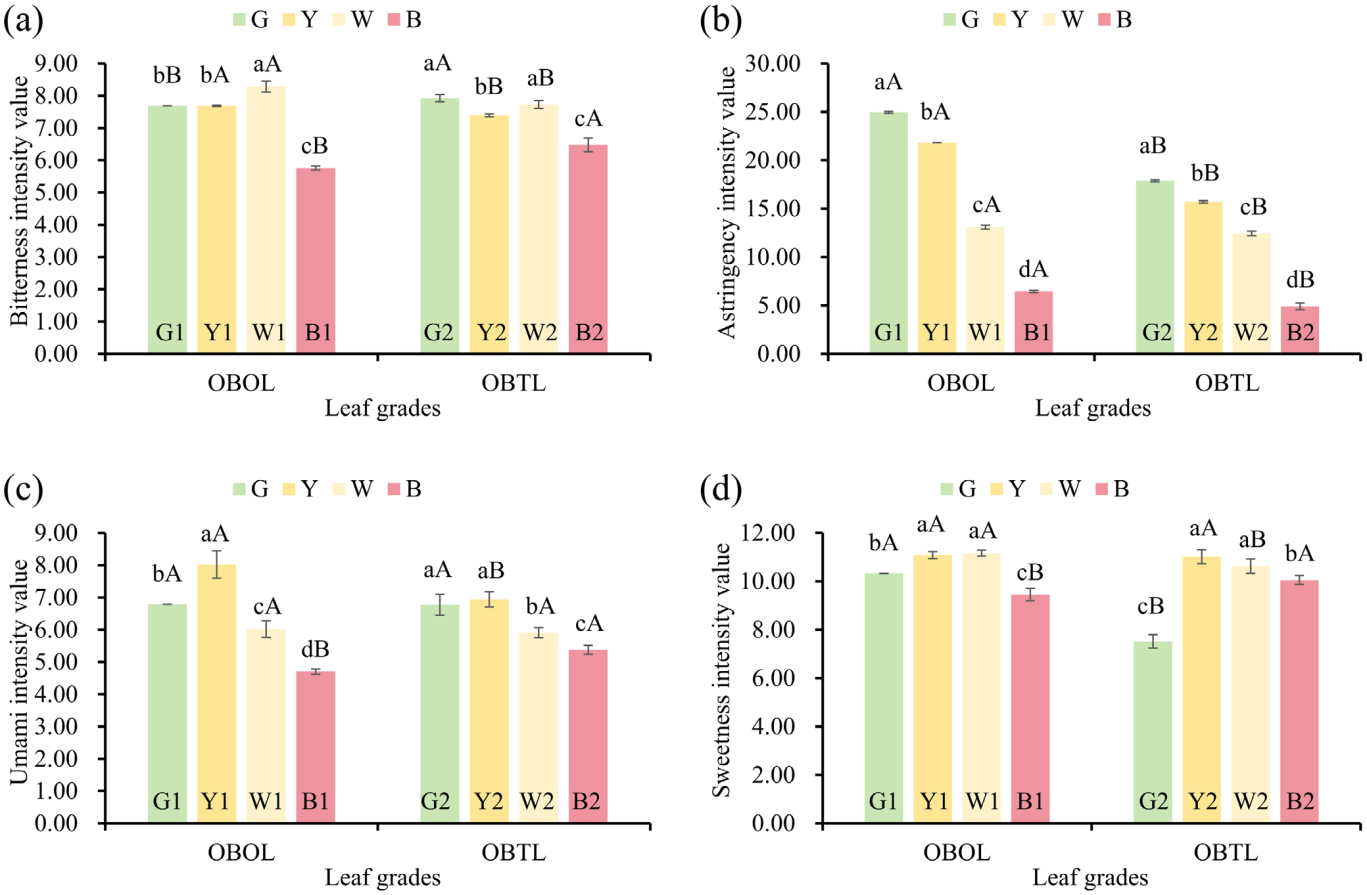
E‐tongue analysis of summer tea processed by four machining methods. Histogram showing the intensity values of bitterness (a), astringency (b), umami (c), and sweetness (d). G1 and G2 represented green tea produced from summer fresh tea leaves with one bud and one leaf, and on one bud with two to three leaves, respectively. Y1 and Y2 represented yellow tea produced from summer fresh tea leaves with one bud and one leaf, and on one bud with two to three leaves, respectively. W1 and W2 represented white tea produced from summer fresh tea leaves with one bud and one leaf, and on one bud with two to three leaves, respectively. B1 and B2 represented black tea produced from summer fresh tea leaves with one bud and one leaf, and on one bud with two to three leaves, respectively. OBOL referred to fresh tea shoots with one bud and one leaf. OBTL referred to fresh tea shoots with one bud and two to three leaves. Lowercase letters denote significant differences in sensory quality among teas from the same fresh leaf grade but different processing methods (*p* < 0.05). Uppercase letters denote significant differences among teas from the same processing method but different fresh leaf grades (*p* < 0.05).

For teas produced using the same processing method, variations in fresh leaf grades resulted in distinct taste characteristics, including bitterness, astringency, umami, and sweetness. Previous studies have demonstrated that bitterness is closely associated with the grade of fresh leaves (Zou et al. 2018). As shown in Figure 3a, the bitterness intensities of G2 and B2 were significantly higher than these of G1and B1, while these of Y1 and W1 were significantly higher than those of Y2 and W2 (*p* < 0.05). These results suggest that higher leaf tenderness may be a key factor contributing to lower bitterness in green and black teas. However, an opposite trend was observed for yellow and white teas, where bitterness intensity increased with leaf tenderness. The overall trend in bitterness intensity among all tea samples was consistent with the findings reported by Chen et al. (2024). As shown in Figure 3b, a significant positive correlation (*p* < 0.05) was observed between astringency intensity and leaf tenderness across all tea samples, indicating that teas produced from less tender leaves exhibited stroger astringency (Chen et al. 2024). Regarding umami intensity (Figure 3c), leaf tenderness of summer tea showed no significant effect on green and white teas, whereas a positive association were observed in yellow and black teas. Sweetness is also a critical sensory attribute contributing to the overall taste balance of tea (Li et al. 2025; Wang et al. 2025). According to the electronic tongue results (Figure 3d), the sweetness intensity of G1 and W1 was significantly higher than that of G2 and W2, respectively. In contrast, an opposite trend was observed in black tea, while no significant effect of leaf tenderness on the sweetness of yellow tea was found.

Overall, the E‐tongue results were consistent with sensory evaluation, providing objective and quantifiable evidence for describing tea sensory attributes (Yang et al. 2024). From the perspective of processing methods, black tea processing effectively reduced the bitterness intensity of summer tea, with B1 (5.76 ± 0.05) and B2 (6.47 ± 0.17) exhibiting the lowest bitterness values among all samples (*p* < 0.05). When analyzed by tea type, the astringency intensity followed the order: green tea > yellow tea > white tea > black tea, while the umami intensity showed a similar trend: green tea > yellow tea > white tea > black tea. These results indicate that processing methods play a critical role in determining tea quality characteristics (Li et al. 2025). Regarding sweetness, no significant difference was observed between yellow and white teas; however, both exhibited significantly higher sweetness intensity than green and black teas (*p* < 0.05). The pronounced bitterness and astringency are long‐recognized quality drawbacks of summer tea (Wen, Sun, Chen, et al. 2023). These findings suggest that producing yellow, white, and black teas from summer tea leaves may be an effective strategy to improve their inherent quality limitations. Leaf tenderness and processing methods jointly regulate the four fundamental taste attributes, while different tea types exhibit distinct responses to raw material grades and processing conditions, playing a critical role in shaping the diverse taste profiles of summer tea.

### 3.3 Targeted Metabolomics Analysis

The flavor of tea is primarily derived from its intrinsic chemical constituents, among which amino acids, catechins, and caffeine are the key taste‐active compounds, mainly contributing to bitterness, astringency, umami, and sweetness (Shen et al. 2025). Specifically, to comprehensively elucidate the effects of summer leaf plucking standards and tea processing methods on taste‐active compounds, a targeted metabolomics approach was employed to quantify 19 amino acids, five catechin components, and caffeine in the tea samples (Table 2). Specifically, Thea, ASP, and Glu contributed to umami taste formation. Ser, Gly, Thr, Ala, and Pro were major contributors to sweetness taste. His, Tyr, Val, Met, Ile, Leu, Phe, CAF, EGC, C, EC, EGCG, and ECG contributed to the bitter taste of tea. EGC, C, EC, EGCG, and ECG contributed to the astringent taste of tea (van Gemert 2011). Compounds that contribute to taste perception were classified into corresponding taste‐related categories, such as EGCG categorized as an astringent compound (Figure 4).

**TABLE 2:** Targeted quantification of taste‐active compounds in four tea types processed from summer tea fresh leaves.

Compounds (mg/g)	Tea Sample							
	G1	G2	Y1	Y2	W1	W2	B1	B2
Thea	9.87 ± 0.11b	13.85 ± 0.04a	8.98 ± 0.10d	9.10 ± 0.07d	4.17 ± 0.01f	4.56 ± 0.04	5.85 ± 0.09e	9.29 ± 0.12c
Asp	1.44 ± 0.01e	1.418 ± 0.00d	1.43 ± 0.02e	1.57 ± 0.01c	1.85 ± 0.03a	1.43 ± 0.00e	1.74 ± 0.00b	1.22 ± 0.01f
Glu	2.74 ± 0.02c	2.66 ± 0.04c	2.17 ± 0.07e	3.66 ± 0.16a	2.97 ± 0.00b	2.39 ± 0.02d	2.50 ± 0.05d	1.88 ± 0.01f
Ser	1.02 ± 0.00c	0.93 ± 0.01e	0.99 ± 0.00d	1.02 ± 0.00c	1.13 ± 0.01a	1.09 ± 0.00b	1.08 ± 0.01b	0.79 ± 0.01f
Gly	0.99 ± 0.02a	0.86 ± 0.01c	0.99 ± 0.00a	0.95 ± 0.04b	0.94 ± 0.00b	0.95 ± 0.00b	0.99 ± 0.03a	0.73 ± 0.00d
Thr	0.91 ± 0.00c	0.80 ± 0.00f	0.99 ± 0.01a	0.93 ± 0.00b	0.88 ± 0.01d	0.85 ± 0.01e	0.91 ± 0.00c	0.63 ± 0.00 g
Ala	0.88 ± 0.00c	0.81 ± 0.00f	0.98 ± 0.01a	0.84 ± 0.00e	0.91 ± 0.01b	0.90 ± 0.00b	0.85 ± 0.00d	0.68 ± 0.00 g
Pro	1.15 ± 0.01a	0.90 ± 0.00e	0.97 ± 0.02d	1.07 ± 0.01b	1.13 ± 0.00a	1.00 ± 0.01c	1.13 ± 0.02a	0.76 ± 0.00f
His	0.36 ± 0.00a	0.34 ± 0.00ab	0.33 ± 0.02ab	0.36 ± 0.02a	0.39 ± 0.07a	0.38 ± 0.01a	0.38 ± 0.02a	0.29 ± 0.00b
Arg	0.94 ± 0.00b	0.73 ± 0.01 g	0.76 ± 0.00f	1.25 ± 0.01a	0.88 ± 0.00d	0.78 ± 0.00e	0.92 ± 0.00c	0.66 ± 0.00 h
Tyr	0.53 ± 0.00a	0.41 ± 0.00c	0.43 ± 0.00c	0.51 ± 0.08a	0.49 ± 0.01ab	0.51 ± 0.01a	0.45 ± 0.00bc	0.31 ± 0.01c
Val	0.94 ± 0.00b	0.80 ± 0.01f	0.82 ± 0.00e	0.90 ± 0.01c	0.96 ± 0.00a	0.84 ± 0.01d	0.96 ± 0.00a	0.67 ± 0.000 g
Met	0.001 ± 0.00a	0.001 ± 0.00a	0.001 ± 0.00a	0.001 ± 0.00a	0.001 ± 0.00a	0.001 ± 0.00a	0.001 ± 0.00a	0.001 ± 0.00a
Ile	0.77 ± 0.00a	0.63 ± 0.00d	0.66 ± 0.00c	0.71 ± 0.00b	0.77 ± 0.00a	0.67 ± 0.01c	0.77 ± 0.00a	0.50 ± 0.01e
Leu	1.42 ± 0.01a	1.25 ± 0.00c	1.22 ± 0.01d	1.36 ± 0.00a	1.37 ± 0.02a	1.32 ± 0.00b	1.37 ± 0.01a	1.03 ± 0.00e
Phe	0.96 ± 0.00bc	0.81 ± 0.01e	0.86 ± 0.04d	0.98 ± 0.01b	1.05 ± 0.00a	0.88 ± 0.00d	0.93 ± 0.02c	0.64 ± 0.01e
CAF	40.34 ± 0.47ab	35.31 ± 0.25c	40.73 ± 0.61ab	34.80 ± 0.66c	40.02 ± 0.40b	29.07 ± 0.05d	41.28 ± 0.85a	28.20 ± 0.16d
EGC	8.10 ± 0.07d	23.21 ± 0.18a	10.51 ± 0.07c	22.22 ± 0.08b	4.29 ± 0.23f	5.43 ± 0.05e	0.99 ± 0.01 h	2.02 ± 0.03 g
C	1.51 ± 0.00c	1.39 ± 0.01 cd	1.11 ± 0.09e	1.31 ± 0.00d	1.36 ± 0.04d	1.12 ± 0.06e	3.82 ± 0.14a	2.61 ± 0.01b
EC	9.63 ± 0.04d	15.13 ± 0.05a	10.57 ± 0.05c	13.37 ± 0.21b	6.97 ± 0.05e	5.71 ± 0.07f	1.01 ± 0.08 h	4.27 ± 0.05 g
EGCG	60.70 ± 2.14a	49.41 ± 2.70c	55.32 ± 1.65b	45.14 ± 1.66d	29.11 ± 0.24e	21.01 ± 0.89f	3.39 ± 0.01 g	4.89 ± 0.01 g
ECG	27.12 ± 0.05a	16.57 ± 0.70c	22.51 ± 1.15b	16.11 ± 0.01c	13.31 ± 0.02d	8.02 ± 0.22e	6.11 ± 0.09f	4.31 ± 0.05 g
Lys	0.94 ± 0.00b	0.79 ± 0.00f	0.82 ± 0.01e	0.92 ± 0.01c	0.97 ± 0.00a	0.91 ± 0.00d	0.93 ± 0.01bc	0.66 ± 0.01 g
Cys	0.09 ± 0.00b	0.08 ± 0.00c	0.08 ± 0.00c	0.10 ± 0.00a	0.07 ± 0.00d	0.07 ± 0.01d	0.05 ± 0.00e	0.03 ± 0.00f
Hyp	0.15 ± 0.00e	0.17 ± 0.00bc	0.16 ± 0.00d	0.18 ± 0.00b	0.17 ± 0.01bc	0.21 ± 0.00a	0.17 ± 0.00 cd	0.22 ± 0.01a

*Note:* G1 and G2 represented green tea produced from summer fresh tea leaves with one bud and one leaf and on one bud with two to three leaves, respectively. Y1 and Y2 represented yellow tea produced from summer fresh tea leaves with one bud and one leaf and on one bud with two to three leaves, respectively. W1 and W2 represented white tea produced from summer fresh tea leaves with one bud and one leaf and on one bud with two to three leaves, respectively. B1 and B2 represented black tea produced from summer fresh tea leaves with one bud and one leaf and on one bud with two to three leaves, respectively. The data were expressed in the form of “mean ± standard” deviation. Lowercase letters indicate significant differences in the contents of taste‐related compounds among all tea samples (*p* < 0.05).

**FIGURE 4:**
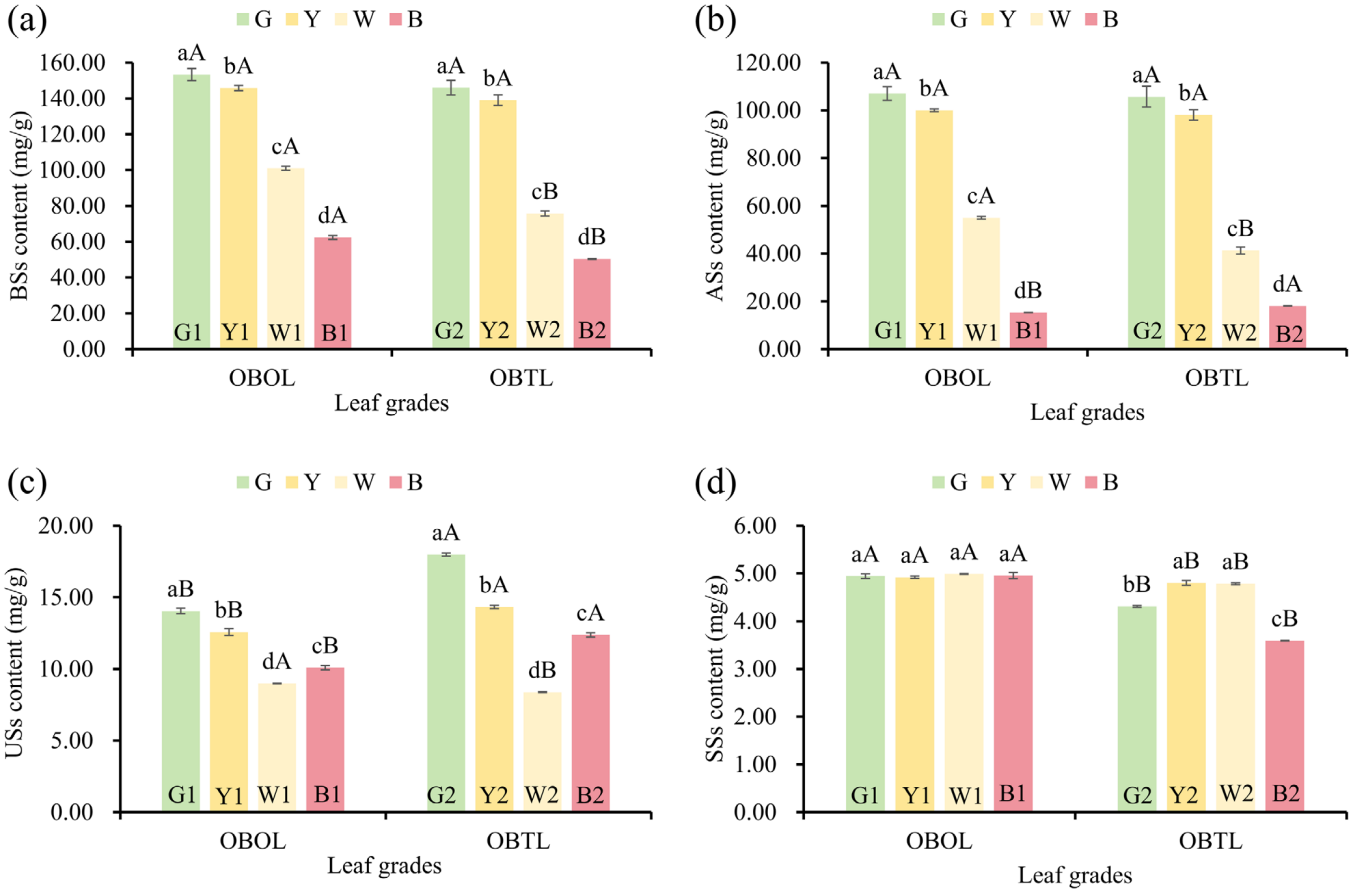
Content of taste‐related compounds in four types of summer tea, including bitterness substances (BSs) (a), astringency substances (ASs) (b), umami substances (Uss) (c), and sweetness substances (SSs) (d). BSs represented bitterness substances, Ass represented astringency substances, Uss represented umami substances, and SSs represented sweetness substances. G1 and G2 represented green tea produced from summer fresh tea leaves with one bud and one leaf, and on one bud with two to three leaves, respectively. Y1 and Y2 represented yellow tea produced from summer fresh tea leaves with one bud and one leaf, and on one bud with two to three leaves, respectively. W1 and W2 represented white tea produced from summer fresh tea leaves with one bud and one leaf, and on one bud with two to three leaves, respectively. B1 and B2 represented black tea produced from summer fresh tea leaves with one bud and one leaf, and on one bud with two to three leaves, respectively. OBOL referred to fresh tea shoots with one bud and one leaf. OBTL referred to fresh tea shoots with one bud and two to three leaves. Lowercase letters denote significant differences in sensory quality among teas from the same fresh leaf grade but different processing methods (*p* < 0.05). Uppercase letters denote significant differences among teas from the same processing method but different fresh leaf grades (*p* < 0.05).

The contents of taste‐active compounds in teas produced from fresh leaves with different tenderness levels were analyzed (Figure 4). The results showed that leaf tenderness had no significant effect on the contents of bitter and astringent compounds in green and yellow teas, whereas significant effects were observed in white and black teas (*p* < 0.05). Specifically, the contents of bitter‐and‐astringent‐related compounds in W1 and B1 were significantly higher than those in W2 and B2 (*p* < 0.05). Regarding umami‐related compounds, G2, Y2, and B2 exhibited significantly higher contents than G1, Y1, and B1, respectively, while W1 showed significantly higher levels than W2 (*p* < 0.05). In addition, a significant positive correlation was observed between leaf tenderness and the contents of sweet compounds (*p* < 0.05). These findings indicated that leaf tenderness plays a critical role in shaping tea taste. This suggested that the sensory perception of bitterness and astringency is not solely determined by their absolute concentrations but was also influenced by processing‐induced chemical transformations and mutual influence effects among taste‐active compounds. Tender summer leaves may be more favorable for white and black tea production, whereas summer leaves with one bud and three leaves contain higher levels of umami compounds, making them more suitable for green and yellow tea processing.

From the perspective of processing effects on taste‐active compounds, distinct trends were observed among the four tea types produced from summer leaves of the same tenderness level. The contents of bitter and astringent compounds followed the order: green tea > yellow tea > white tea > black tea (Figure 4), indicating that processing methods significantly influenced the degradation and transformation (*p* < 0.05). In contrast, the contents of umami‐related compounds exhibited a different pattern, following the order: green tea > yellow tea > black tea > white tea. This suggests that processing methods exert differential effects on the retention and amino acid retention and conversion. Except for G2 and B2, no significant differences were observed in sweetness‐related compounds among tea types produced from the same leaf grade (*p* < 0.05). These differences may be attributed to the distinct biochemical reactions occurring during processing, including enzyme inactivation, oxidation, hydrolysis, and thermal degradation.

Based on the above analysis, a negative association was observed between leaf tenderness and the contents of astringent compounds. Under high‐temperature conditions in summer, tender leaves exhibit enhanced metabolic activity, while the degradation of bitterness‐related and astringent‐related compounds is accelerated in more mature leaves (one bud with three leaves) (Li et al. 2018). Therefore, selecting relatively mature fresh leaves for summer tea processing may effectively alleviate the undesirable astringency. In addition, yellow tea processing can effectively reduce the contents of bitter and astringent compounds (Tu et al. 2025; Wei et al. 2022). This reduction is primarily attributed to the oxidation and transformation of polyphenolic compounds under the moist‐heat conditions of the yellowing process, leading to decreased levels of bitterness‐ and astringency‐related compounds (Xu et al. 2018).

### 3.4 Screening of Key Taste‐Active Compounds Based on TAVs

The taste activity values (TAVs) evaluated the contribution of individual taste‐active compounds to the sensory attributes in tea samples derived from summer‐harvested leaves. The non‐volatile compounds with TAVs > 1 were considered as the key taste compounds of the taste of tea samples. And higher TAVs indicated greater potential contributions to taste perception. Based on the taste threshold values of 22 non‐volatile compounds with taste‐active properties in tea (Yang et al. 2024), their taste activity values (TAVs) were calculated. A total of 10 key taste‐active compounds with TAVs > 1 were identified, including Asp, Glu, Thea, Thr, His, Arg, Val, Phe, CAF, and EGCG. Among these, Asp, Glu, and Thea mainly contribute to umami taste. Thea contributed to sweetness, and His, Arg, Val, and Phe were associated with bitterness, while EGCG exhibits both bitter and astringent characteristics (van Gemert 2011).

As shown in Figure 5 and Table 3, Thea content was significantly higher in teas made from one bud with three leaves than from one bud with one leaf (*p* < 0.05), likely due to the higher proportion of tender stems in the former. Thea, the most abundant amino acid in tea infusion, can be enhanced by tender stems (Deka et al. 2024; Lin et al. 2022). Glu content did not differ significantly in green tea, but in other tea types it followed Y2 > Y1 and W1, B1 > W2, B2 (*p* < 0.05). Asp content was higher in G2 and Y2 than in G1 and Y1, while W1 and B1 exceeded W2 and B2 (*p* < 0.05). For summer tea, greater leaf tenderness favored Thea, Glu, and Asp accumulation in white and black teas, whereas more mature leaves with abundant tender stems were better suited for enhancing Glu and Asp in green and yellow teas. Thr content was higher in one‐bud‐one‐leaf teas (*p* < 0.05), indicating that tenderness is linked to increased sweetness. His, Arg, Val, and Phe showed consistent patterns: G1, B1 > G2, B2; Y2 > Y1; and W1 > W2 (*p* < 0.05), suggesting that bitter amino acid accumulation correlates with tenderness, possibly due to protein degradation under high temperature and UV radiation (Ma et al. 2022). Black tea processing reduced bitter compounds and improved flavor. EGCG levels followed G1, Y1, W1 > G2, Y2, W2, but B2 > B1 (*p* < 0.05), indicating a positive correlation with tenderness in unfermented and lightly fermented teas (green, yellow, white), while this correlation was not consistent in black tea owing to oxidative transformation during high‐temperature fermentation.

**FIGURE 5:**
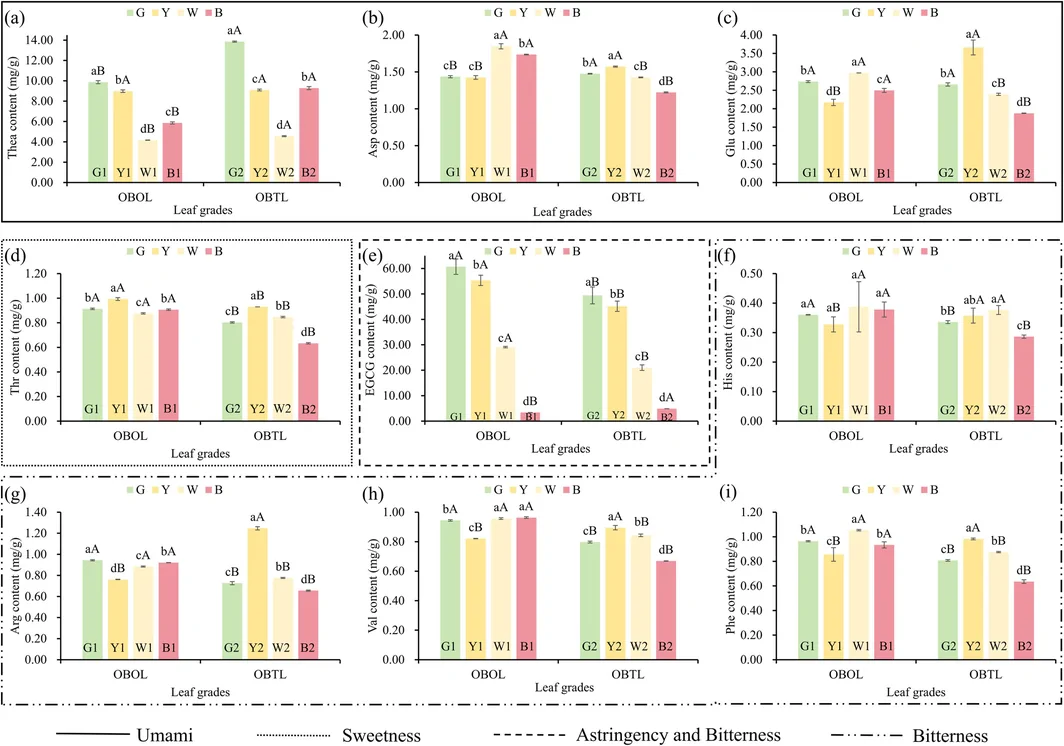
TAVs of taste‐related compounds in four types of summer tea. (a–c) umami taste‐related compounds; (d) sweet taste‐related compounds; (e) astringent taste‐related compounds; (f–i) bitterness taste‐related compounds. G1 and G2 represented green tea produced from summer fresh tea leaves with one bud and one leaf, and on one bud with two to three leaves, respectively. Y1 and Y2 represented yellow tea produced from summer fresh tea leaves with one bud and one leaf, and on one bud with two to three leaves, respectively. W1 and W2 represented white tea produced from summer fresh tea leaves with one bud and one leaf, and on one bud with two to three leaves, respectively. B1 and B2 represented black tea produced from summer fresh tea leaves with one bud and one leaf, and on one bud with two to three leaves, respectively. OBOL referred to fresh tea shoots with one bud and one leaf. OBTL referred to fresh tea shoots with one bud and two to three leaves. Lowercase letters denote significant differences in sensory quality among teas from the same fresh leaf grade but different processing methods (*p* < 0.05). Uppercase letters denote significant differences among teas from the same processing method but different fresh leaf grades (*p* < 0.05).

**TABLE 3:** TAVs analysis of taste‐active compounds in four tea types processed from summer tea fresh leaves.

Taste	Taste compounds	Taste threshold (mg/g)	TAVs							
			G1	Y1	W1	B1	G2	Y2	W2	B2
Umami	ASP	1	1.44 ± 0.01	1.43 ± 0.02	1.85 ± 0.03	1.74 ± 0.00	1.48 ± 0.00	1.57 ± 0.01	1.43 ± 0.00	1.22 ± 0.01
	Glu	0.3	9.13 ± 0.07	7.23 ± 0.23	9.90 ± 0.00	8.33 ± 0.15	8.87 ± 0.12	12.20 ± 0.55	7.97 ± 0.08	6.27 ± 0.02
	Thea	0.06	164.50 ± 2.05	149.67 ± 1.70	69.50 ± 0.22	97.50 ± 1.43	230.83 ± 0.75	151.67 ± 1.16	76.00 ± 0.67	154.83 ± 2.08
Sweet	Ser	1.5	0.68 ± 0.00	0.66 ± 0.00	0.75 ± 0.00	0.72 ± 0.00	0.62 ± 0.01	0.68 ± 0.00	0.73 ± 0.00	0.53 ± 0.01
	Gly	2.6	0.38 ± 0.01	0.38 ± 0.00	0.36 ± 0.00	0.38 ± 0.01	0.33 ± 0.00	0.37 ± 0.01	0.37 ± 0.00	0.28 ± 0.00
	Thr	0.6	1.52 ± 0.01	1.65 ± 0.01	1.47 ± 0.01	1.52 ± 0.01	1.33 ± 0.01	1.55 ± 0.00	1.42 ± 0.01	1.05 ± 0.01
	Ala	3	0.29 ± 0.00	0.33 ± 0.00	0.30 ± 0.00	0.28 ± 0.00	0.27 ± 0.00	0.28 ± 0.00	0.30 ± 0.00	0.23 ± 0.00
	Pro	1.3	0.88 ± 0.01	0.75 ± 0.01	0.87 ± 0.00	0.87 ± 0.02	0.69 ± 0.00	0.82 ± 0.00	0.77 ± 0.00	0.58 ± 0.00
Bitter	His	0.2	1.80 ± 0.00	1.65 ± 0.10	1.95 ± 0.35	1.90 ± 0.10	1.70 ± 0.02	1.80 ± 0.10	1.90 ± 0.06	1.45 ± 0.02
	Arg	0.5	1.88 ± 0.01	1.52 ± 0.00	1.76 ± 0.01	1.84 ± 0.00	1.46 ± 0.02	2.50 ± 0.03	1.56 ± 0.01	1.32 ± 0.01
	Tyr	0.73	0.73 ± 0.01	0.59 ± 0.01	0.67 ± 0.01	0.62 ± 0.01	0.56 ± 0.01	0.70 ± 0.11	0.70 ± 0.01	0.42 ± 0.00
	Val	0.4	2.35 ± 0.01	2.05 ± 0.00	2.40 ± 0.01	2.40 ± 0.01	2.00 ± 0.01	2.25 ± 0.03	2.11 ± 0.02	1.68 ± 0.00
	Met	0.3	0.00 ± 0.00	0.00 ± 0.00	0.00 ± 0.00	0.00 ± 0.00	0.00 ± 0.00	0.00 ± 0.00	0.00 ± 0.00	0.00 ± 0.00
	Ile	0.9	0.86 ± 0.00	0.73 ± 0.00	0.86 ± 0.00	0.86 ± 0.01	0.70 ± 0.00	0.79 ± 0.00	0.74 ± 0.01	0.56 ± 0.01
	Leu	1.9	0.75 ± 0.01	0.64 ± 0.01	0.72 ± 0.01	0.72 ± 0.00	0.66 ± 0.00	0.72 ± 0.00	0.69 ± 0.00	0.54 ± 0.00
	Phe	0.9	1.07 ± 0.00	0.96 ± 0.05	1.17 ± 0.01	1.03 ± 0.02	0.9 ± 0.01	1.09 ± 0.01	0.98 ± 0.00	0.71 ± 0.01
	C	70.36	0.02 ± 0.00	0.02 ± 0.00	0.02 ± 0.00	0.05 ± 0.00	0.02 ± 0.00	0.02 ± 0.00	0.02 ± 0.00	0.04 ± 0.00
	EGCG	28.41	2.14 ± 0.09	1.95 ± 0.06	1.02 ± 0.01	0.12 ± 0.00	1.74 ± 0.10	1.59 ± 0.06	0.74 ± 0.03	0.17 ± 0.00
	EC	70.4	0.14 ± 0.00	0.15 ± 0.00	0.10 ± 0.00	0.01 ± 0.00	0.21 ± 0.00	0.19 ± 0.00	0.08 ± 0.00	0.06 ± 0.00
	EGC	22.46	0.36 ± 0.00	0.47 ± 0.00	0.19 ± 0.01	0.04 ± 0.00	1.03 ± 0.01	0.99 ± 0.00	0.24 ± 0.00	0.09 ± 0.00
	ECG	140.78	0.19 ± 0.00	0.16 ± 0.01	0.09 ± 0.00	0.04 ± 0.00	0.12 ± 0.00	0.11 ± 0.00	0.06 ± 0.00	0.03 ± 0.00
	CAF	25	1.61 ± 0.02	1.41 ± 0.01	1.63 ± 0.02	1.39 ± 0.03	1.60 ± 0.02	1.16 ± 0.00	1.65 ± 0.03	1.13 ± 0.01
Astringent	EC	76.13	0.13 ± 0.00	0.14 ± 0.00	0.09 ± 0.00	0.01 ± 0.00	0.20 ± 0.00	0.18 ± 0.00	0.08 ± 0.00	0.06 ± 0.00
	EGC	44.91	0.18 ± 0.00	0.23 ± 0.00	0.10 ± 0.01	0.02 ± 0.00	0.52 ± 0.00	0.49 ± 0.00	0.12 ± 0.00	0.04 ± 0.00
	ECG	32.44	0.84 ± 0.00	0.69 ± 0.04	0.41 ± 0.00	0.19 ± 0.00	0.51 ± 0.02	0.50 ± 0.00	0.25 ± 0.01	0.13 ± 0.00
	EGCG	24.56	2.47 ± 0.10	2.25 ± 0.07	1.19 ± 0.01	0.14 ± 0.00	2.01 ± 0.11	1.84 ± 0.07	0.86 ± 0.04	0.20 ± 0.00

*Note:* G1 and G2 represented green tea produced from summer fresh tea leaves with one bud and one leaf and one bud with two to three leaves, respectively. Y1 and Y2 represented yellow tea produced from summer fresh tea leaves with one bud and one leaf and one bud with two to three leaves, respectively. W1 and W2 represented white tea produced from summer fresh tea leaves with one bud and one leaf and one bud with two to three leaves, respectively. B1 and B2 represented black tea produced from summer fresh tea leaves with one bud and one leaf and one bud with two to three leaves, respectively.

Regarding processing methods, Thea content varied distinctly among tea types from different raw materials. For one‐bud‐one‐leaf teas, the order was green tea > yellow tea > black tea > white tea; for one‐bud‐three‐leaf teas, it was green tea > black tea > yellow tea > white tea. White and yellow tea processing helped retain Asp, Glu, and Phe, likely due to mild fermentation and withering that better preserve amino acids, indicating that processing methods are key determinants of umami‐related compound levels. Thr content was highest in yellow teas (Y1 and Y2) (*p* < 0.05), suggesting yellow tea processing enhances sweetness in summer tea. High tenderness did not significantly affect His across tea types, while B2 showed the lowest His (*p* < 0.05). Additionally, yellow and white tea processing reduced Arg and Val in summer tea (*p* < 0.05). EGCG followed a consistent order across all samples: green tea > yellow tea > white tea > black tea (*p* < 0.05) (Jiang et al. 2019), indicating direct regulation by processing methods. These findings suggest that highly fermented processing effectively mitigates the undesirable bitterness and astringency of summer tea.

Based on the above results, it was found that summer tea leaf plucking standards and processing methods exhibited combined effects on the accumulation of taste‐active compounds, particularly on Thea and EGCG. Thea, the most abundant amino acid in tea, is mainly associated with the umami taste, whereas EGCG, a major catechin in tea, contributes mainly to astringency. In general, increased Thea levels and reduced EGCG contents are considered key factors for enhancing the freshness and palatability of tea infusion (Yu et al. 2025; Zhang et al. 2020). Taken together, the use of more mature summer tea leaves (one bud with three leaves) combined with highly fermented processing methods is conducive to reducing astringency in summer tea, thereby improving its overall sensory quality. This provides important implications for the quality optimization and high‐value utilization of summer tea resources.

### 3.5 Volatile Metabolites Analysis

HS‐SPME‐GC–MS analysis identified a total of 1619 volatile metabolites in 4 types of tea produced from summer leaves of one bud with one leaf and one bud with three leaves (Table S2). These metabolites were classified into 15 categories (Figure 6a), including 314 terpenoids (19.39%), 289 esters (17.85%), 163 heterocyclic compounds (10.07%), 141 alcohols (8.71%), 181 ketones (11.18%), 112 aldehydes (6.92%), 52 aromatic hydrocarbons (3.21%), 100 hydrocarbons (6.18%), 73 acids (4.51%), 28 nitrogen‐containing compounds (1.73%), 63 phenolic compounds (3.89%), 6 sulfur‐containing compounds (0.37%), 59 amines (3.64%), 28 ethers (1.73%), and 10 halogenated hydrocarbons (0.62%).

**FIGURE 6:**
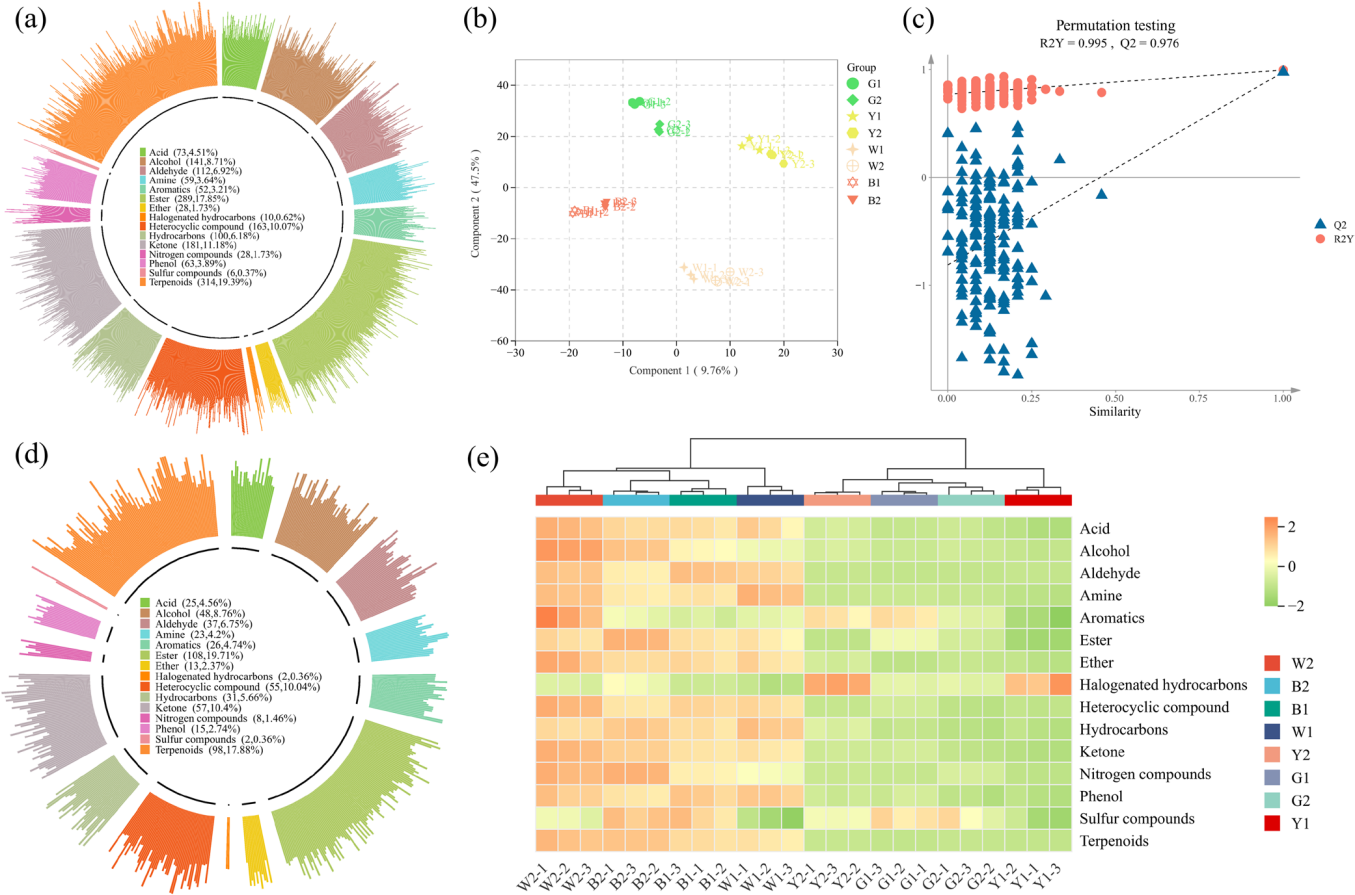
Volatile metabolites analysis of the four types of summer tea. (a) pie chart of the overall composition of volatile metabolites in four types of summer tea; (b) OPLS‐DA analysis of volatile compound profiles; (c) a permutation test was performed to validate the OPLS‐DA model; (d) pie chart of the composition of the DMVs in four types of summer tea; (e) heatmap analysis of the DMVs in four types of summer tea. G1 and G2 represented green tea produced from summer fresh tea leaves with one bud and one leaf, and on one bud with two to three leaves, respectively. Y1 and Y2 represented yellow tea produced from summer fresh tea leaves with one bud and one leaf, and on one bud with two to three leaves, respectively. W1 and W2 represented white tea produced from summer fresh tea leaves with one bud and one leaf, and on one bud with two to three leaves, respectively. B1 and B2 represented black tea produced from summer fresh tea leaves with one bud and one leaf, and on one bud with two to three leaves, respectively.

To further investigate the effects of plucking standards and processing methods on volatile metabolites in multiple tea types derived from summer fresh leaves, orthogonal partial least squares discriminant analysis (OPLS‐DA) was performed (Figure 6b). The OPLS‐DA model demonstrated clear separation of the four tea types produced under different plucking standards into distinct quadrants, with low intra‐group variability and pronounced inter‐group discrimination, while samples of the same tea type were well clustered together. A 200‐times permutation test (*p* < 0.01) showed that the intercept of the regression lines for *Q* ^2^ (*Q* ^2^ = −0.976) was below zero, indicating that the model had no obvious overfitting (Figure 6c).

These results indicate that processing methods exerted a greater influence on volatile profiles than plucking standards. Differential volatile metabolites (DVMs) were screened based on the criteria of VIP > 1, fold change ≥ 2 or ≤ 0.5, and *p* < 0.05. A total of 548 DVMs were identified (Table S3), mainly consisting of 98 terpenoids (17.88%), 108 esters (19.71%), 55 heterocyclic compounds (10.04%), 48 alcohols (8.76%), and 57 ketones (10.40%) (Figure 6d). Hierarchical clustering heatmap analysis of DVMs across different tea infusions (Figure 6e) revealed that tea processing methods exerted a greater influence on the composition and abundance of DVMs than plucking standards of summer fresh leaves. According to the clustering results, green tea and yellow tea were grouped into one cluster, while white tea and black tea formed another cluster. Moreover, the overall abundance of DVMs in white and black teas was significantly higher than that in green and yellow teas. These findings indicate that processing summer tea leaves into white and black teas results in superior aroma quality compared to green and yellow teas, which may help to overcome the limitation of low aroma richness commonly associated with summer tea (Liang et al. 2024; Liu et al. 2023).

### 3.6 Identification of Key Volatile Metabolites

As a quantitative evaluation method, rOAVs were based on the odor thresholds of volatile metabolites and are widely used to identify key aroma‐active compounds in foods. Generally, compounds with rOAV ≥ 1 are considered perceptible and have a significant impact on the overall flavor, with higher rOAV values indicating a greater contribution. Based on the odor thresholds reported in previous studies (van Gemert 2011), a total of 126 key volatile metabolites (KMVs) with rOAV ≥ 1 were identified. These included 24 terpenoids (19.05%), 19 ketones (15.08%), 17 esters (13.49%), 17 aldehydes (13.49%), 14 heterocyclic compounds (11.11%), 12 alcohols (9.52%), 6 aromatic hydrocarbons (4.76%), 6 phenolic compounds (4.76%), 4 ethers (3.17%), 4 nitrogen‐containing compounds (3.17%), 2 amines (1.59%), and 1 sulfur‐containing compound (0.79%) (Table S4).

To further elucidate the contribution of key volatile metabolites (KMVs) to the aroma of summer tea, 10 KMVs with rOAV ≥ 1000 were selected for detailed analysis from the tea samples, including geraniol (rOAV = 20.11–1690.27), (Z)‐4‐heptenal (rOAV = 360.95–4376.40), 3‐methyl‐2‐phenylethyl ester‐butanoic acid (rOAV = 535.25–3812.73), (E,E)‐3,5‐octadien‐2‐one (rOAV = 133.35–8488.72), 1‐octen‐3‐one (rOAV = 1439.48–8445.30), dimethyl trisulfide (rOAV = 473.72–11300.43), dihydro‐2‐methyl‐3(2H)‐furanone (rOAV = 62,774.11–340,773.63), (E)‐4‐nonenal (rOAV = 579.36–10,482.90), 1,3,5‐trithiane (rOAV = 19.22–2607.80), and 1‐hexen‐3‐one (rOAV = 3003.67–26,859.76) (Figure 7). These compounds play important roles in shaping the aroma characteristics of the four tea types (green, yellow, white, and black teas) produced from summer tea leaves. Notably, dihydro‐2‐methyl‐3(2H)‐furanone exhibited the highest rOAVs across all samples, suggesting that it may serve as a key characteristic aroma compound in summer tea.

**FIGURE 7:**
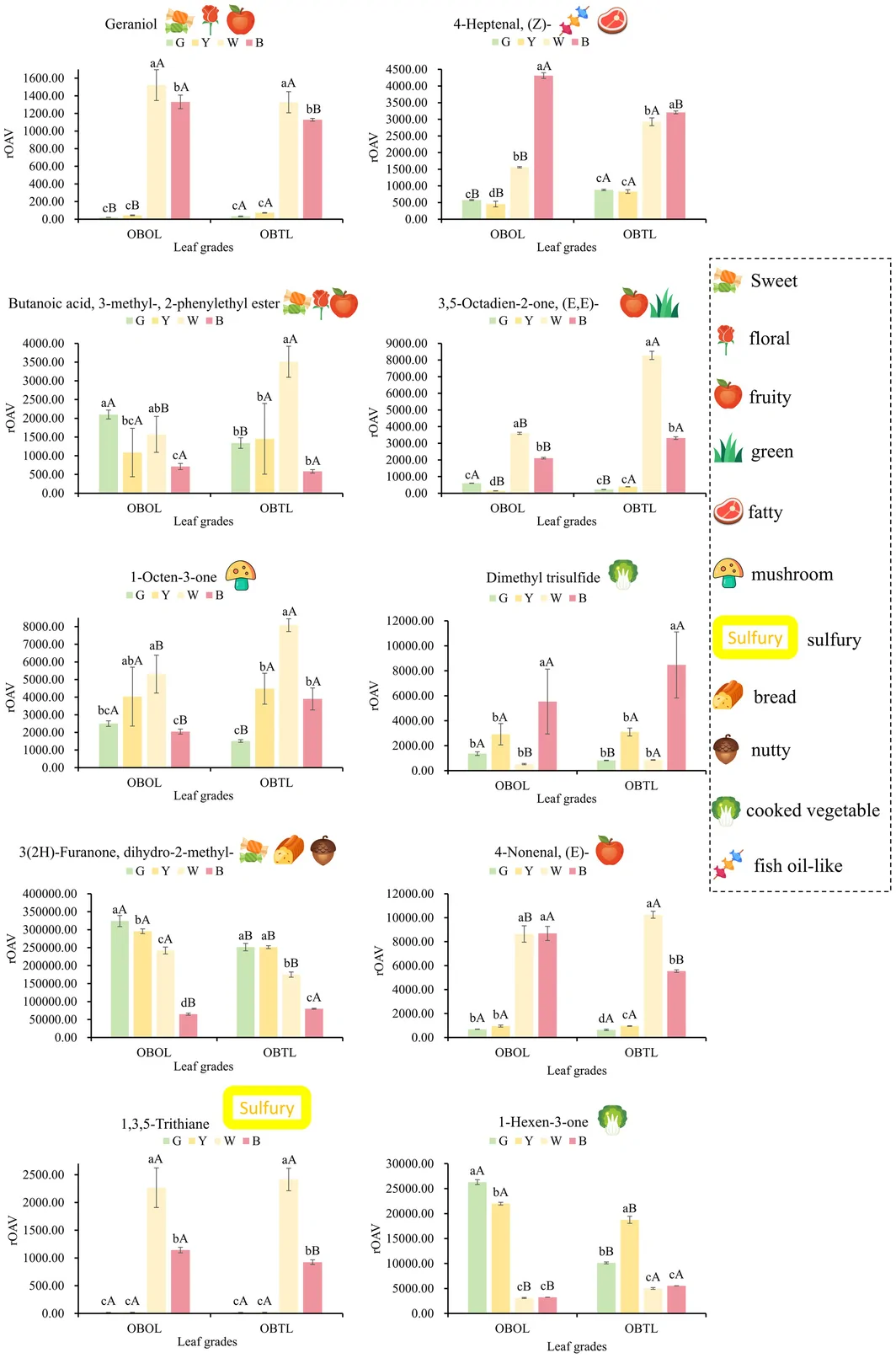
Key volatile compounds (KMVs) identified in four types of summer tea. G1 and G2 represented green tea produced from summer fresh tea leaves with one bud and one leaf, and on one bud with two to three leaves, respectively. Y1 and Y2 represented yellow tea produced from summer fresh tea leaves with one bud and one leaf, and on one bud with two to three leaves, respectively. W1 and W2 represented white tea produced from summer fresh tea leaves with one bud and one leaf, and on one bud with two to three leaves, respectively. B1 and B2 represented black tea produced from summer fresh tea leaves with one bud and one leaf, and on one bud with two to three leaves, respectively.

Analysis of volatile profiles in four summer tea types from different plucking standards revealed distinct variations in key aroma‐active compounds. Geraniol and (Z)‐4‐heptenal showed higher rOAVs in B1 than B2, whereas in green and yellow teas, G2 and Y2 had higher rOAVs than G1 and Y1, respectively. In green tea, G1 exceeded G2 in rOAVs of 2‐phenylethyl 3‐methylbutanoate, dimethyl trisulfide, (E,E)‐3,5‐octadien‐2‐one, and 1‐octen‐3‐one. However, for (E,E)‐3,5‐octadien‐2‐one in yellow, white, and black teas, rOAVs were higher in Y2, W2, and B2 than in Y1, W1, and B1. W2 also showed higher rOAVs than W1 for (Z)‐4‐heptenal, 2‐phenylethyl 3‐methylbutanoate, and dimethyl trisulfide. rOAVs of 1‐octen‐3‐one were higher in W2 and B2 than in W1 and B1. Dihydro‐2‐methyl‐3(2H)‐furanone followed G1, Y1, W1 > G2, Y2, W2, and B2 > B1. For (E)‐4‐nonenal, trends were B1 > B2 and W2 > W1. B1 exceeded B2 in rOAV of 1,3,5‐trithiane. 1‐Hexen‐3‐one showed G1, Y1 > G2, Y2, while W2, B2 > W1, B1.

From the perspective of different tea processing methods, geraniol rOAVs followed: white tea > black tea > yellow tea > green tea. Geraniol, with sweet, floral, and fruity notes, is a key tea volatile (Chen, Shi, et al. 2025; Ma et al. 2023; Wu et al. 2022). This suggested that fixation and fermentation levels during processing significantly influence the formation and retention of geraniol. In addition, (Z)‐4‐Heptenal rOAVs followed: black tea > white tea > green tea > yellow tea. It was associated with fatty and fish oil‐like aromas and is considered a key aroma compound in white and black teas, contributing to their delicate and complex flavor profiles (Huang et al. 2022; Wu et al. 2022). 2‐Phenylethyl 3‐methylbutanoate, with sweet, floral, and fruity notes, showed the highest rOAV in W2, indicating its key role in white tea aroma, consistent with Chen, Que, et al. (2025). (E,E)‐3,5‐Octadien‐2‐one, characterized by fruity and green notes, is a key aroma‐active compound in green, yellow, white, and Pu‐erh teas, playing an important role in tea aroma formation (Lin et al. 2025; Shi et al. 2021; Wang et al. 2025; Zhu et al. 2018). In the present study, its rOAVs were significantly highest in white tea (W1 and W2), followed by black tea (B1 and B2). 1‐Octen‐3‐one, which imparts a mushroom‐like aroma (Murray et al. 2020), exhibited the highest rOAV in W1 and W2 compared with other tea samples. Dimethyl trisulfide, associated with a cooked cabbage‐like odor, showed the highest rOAV in black tea (B1 and B2), indicating its importance as a key volatile compound in black tea aroma (Liu et al. 2021). Dihydro‐2‐methyl‐3(2H)‐furanone, characterized by sweet, bread‐like, and nutty notes (Córdoba et al. 2021), exhibited the highest rOAV among all KMVs identified in this study. Its rOAVs followed the trend: green tea > yellow tea > white tea > black tea, suggesting that it may serve as an important aroma contributor in lightly fermented teas such as green and yellow teas derived from summer leaves. (E)‐4‐Nonenal, with a strong fruity aroma (Gao et al. 2025), showed relatively high rOAVs in white tea (W1 and W2) and black tea (B1 and B2). 1,3,5‐Trithiane, characterized by a sulfury odor, is reported as a key aroma compound in *Butyriboletus roseoflavus* (Song et al. 2025). In this study, it exhibited the highest rOAVs in white tea (W1 and W2) and also relatively high levels in black tea (B1 and B2). 1‐Hexen‐3‐one, associated with cooked vegetable‐like aroma (https://www.chemicalbook.com/), showed higher rOAVs in green (G1, G2) and yellow teas (Y1, Y2), indicating its contribution to their aroma profiles.

In summary, the quality of tea produced from summer tea leaves was jointly determined by both plucking standards and processing methods. Fresh leaves with higher tenderness tend to exhibit more pronounced fresh and green aroma characteristics, whereas more mature leaves are associated with richer, fuller floral and fruity flavor profiles. From the perspective of processing methods, white tea and black tea produced from summer tea leaves contained higher levels of key volatile metabolites (KMVs) than green and yellow teas. This facilitates the formation of more intense floral and fruity aromas, thereby enhancing the overall flavor quality of summer tea.

## 4 Conclusion

In this study, sensory omics and chemometric approaches were comprehensively applied to systematically elucidate the combined effects of plucking standards and processing methods on the quality of summer tea leaves. The results demonstrated that plucking standards were closely associated with the sensory performance of tea. Tender leaves (one bud with one leaf) generally exhibited superior freshness, aroma richness, and sweetness, whereas relatively mature leaves (one bud with three leaves) showed greater potential when combined with appropriate processing methods.

Different processing methods showed distinct capacities for improving summer tea quality. Black tea processing effectively reduced bitterness, whereas white and yellow tea processing enhanced sweetness and umami attributes. White and black tea processing promoted the accumulation of volatile metabolites and significantly improved flavor complexity, indicating their potential for high‐value utilization of summer tea resources. These findings suggest that appropriate processing strategies can partially compensate for the quality limitations associated with less tender summer tea leaves. Therefore, tender materials (e.g., one bud with one leaf) are recommended for producing high‐quality summer tea, while suitable processing strategies can be adopted to improve the flavor quality of relatively mature leaves.

Based on TAV and rOAV analyzes, key taste‐active and aroma‐active compounds were identified and quantified, revealing the chemical basis underlying flavor differences among summer teas. Overall, this study highlights the importance of coordinating fresh leaf selection with processing technology for optimizing summer tea quality and provides practical strategies for the high‐value utilization of summer tea resources. Future studies should further elucidate the dynamic transformation mechanisms of key flavor compounds during processing using quantitative and isotope‐labeling approaches.

## Author Contributions

**Jinlong Luo:** conceptualization, methodology, software, formal analysis, investigation, data curation, visualization, writing – original draft. **Siyu Liao:** formal analysis, investigation, data curation, visualization. **Fengjiao Ding:** software, investigation. **Yuqiao Dai:** investigation, data curation. **Zhongying Liu:** software, investigation. **Tuo Zhang:** formal analysis, investigation. **Ting Yang:** data curation, visualization. **Qin Li:** formal analysis, investigation. **Shimao Fang:** conceptualization, methodology. **Yan Li:** software, formal analysis. **Wanping Fang:** writing – review and editing. **Qiang Shen:** conceptualization, writing – review and editing, funding acquisition.

## Funding

This work was supported by Guizhou Major Science and Technology Project (QKH Major [2025]046), Guizhou Provincial Science and Technology Innovation Platform Construction Project (Qian Ke He Platform JSZX [2025]017), Guizhou Academy of Agricultural Sciences Youth Fund Project (Qiannongke General Fund [2024]13), Guizhou Science and Technology Cooperation Project (Qian MS [2025]296), Guizhou Province Basic Research Program (Natural Science) General Project (MS(2025)295), Guizhou Academy of Agricultural Sciences “expose the list to lead” project (JBGS[2024]04), Guizhou Province Tea industry technology system (GZCYCYJSTX‐05), Research on key technologies of digital matching of main characteristic agricultural products in Guizhou (Qian Nongke GJHX [2025]06), and Zunyi Municipal Bureau of Industry and Science Technology Project (KCTD093).

## Conflicts of Interest

The authors declare no conflicts of interest.

## Supporting information


**Table S1:** The intensity values of E‐tongue of summer teas processed by four tea processing method.
**Table S2:** The result of HS‐SPME‐GC–MS analysis of summer teas processed by four tea processing method.
**Table S3:** Differential volatile metabolites (DVMs) determined in four types of summer tea.
**Table S4:** Volatile compounds with rOAV ≥ 1 identified in four types of summer tea.

## Data Availability

The data that supports the findings of this study are available in the Supporting Information of this article.

## References

[fsn372445-bib-0001] Chen, J. , Y. Que , X. Lin , et al. 2025. “Analysis of Volatile Aroma Component Differences in Fuding Gardenia‐Scented White Tea With Different Scenting Years Based on HS‐SPME‐GC‐MS Technology.” Food Industry Technology. 10.13386/j.issn1002-0306.2025010191.

[fsn372445-bib-0002] Chen, L. , Y. Shi , J. Sun , et al. 2025. “Deciphering the Flavor Profile and Seasonal Variation of Black Tea Processed From Cultivar ‘Baiye 1’.” Food Research International 208: 116181. 10.1016/j.foodres.2025.116181. 40263789

[fsn372445-bib-0003] Chen, R. , X. Lai , S. Wen , et al. 2024. “Analysis of Tea Quality of Large‐Leaf Black Tea With Different Harvesting Tenderness Based on Metabolomics.” Food Control 163: 110474. 10.1016/j.foodcont.2024.110474.

[fsn372445-bib-0004] Córdoba, N. , F. L. Moreno , C. Osorio , S. Velásquez , M. Fernandez‐Alduenda , and Y. Ruiz‐Pardo . 2021. “Specialty and Regular Coffee Bean Quality for Cold and Hot Brewing: Evaluation of Sensory Profile and Physicochemical Characteristics.” LWT 145: 111363. 10.1016/j.lwt.2021.111363.

[fsn372445-bib-0005] Deka, H. , P. P. Sarmah , B. Nath , M. Gogoi , S. Datta , and S. Sabhapondit . 2024. “Unveiling the Effect of Leaf Maturity on Biochemical Constituents and Quality of CTC Black Tea: Insights From Northeast India's Commercial Cultivars.” Journal of Food Measurement and Characterization 18, no. 12: 9921–9937. 10.1007/s11694-024-02930-y.

[fsn372445-bib-0006] Ding, F. , Z. Liu , J. Luo , et al. 2025. “Dynamic Evolution and Formation Mechanisms of Aroma Compounds in Qianmei 502 Black Tea During Processing: Insights From Transcriptomic and Metabolomic Analyses.” LWT 228: 118124. 10.1016/j.lwt.2025.118124.

[fsn372445-bib-0007] Fan, F.‐Y. , C.‐S. Huang , Y.‐L. Tong , et al. 2021. “Widely Targeted Metabolomics Analysis of White Peony Teas With Different Storage Time and Association With Sensory Attributes.” Food Chemistry 362: 130257. 10.1016/j.foodchem.2021.130257. 34118510

[fsn372445-bib-0008] Fang, Q.‐T. , W.‐W. Luo , Y.‐N. Zheng , et al. 2022. “Identification of Key Aroma Compounds Responsible for the Floral Ascents of Green and Black Teas From Different Tea Cultivars.” Molecules 27, no. 9: 2809. 10.3390/molecules27092809. PMC9100887 35566160

[fsn372445-bib-0009] Feng, J. , J. Zhuang , Q. Chen , et al. 2024. “The Effect of Maturity of Tea Leaves and Processing Methods on the Formation of Milky Flavor in White Tea—A Metabolomic Study.” Food Chemistry 447: 139080. 10.1016/j.foodchem.2024.139080. 38520904

[fsn372445-bib-0010] Fu, Z. , L. Chen , S. Zhou , Y. Hong , X. Zhang , and H. Chen . 2024. “Analysis of Differences in the Accumulation of Tea Compounds Under Various Processing Techniques, Geographical Origins, and Harvesting Seasons.” Food Chemistry 430: 137000. 10.1016/j.foodchem.2023.137000. 37531914

[fsn372445-bib-0011] Gao, Q. , C. Yang , H. Liu , and S. Wang . 2025. “Enhancement of Highland Barley Nutrition and Flavor via Liquid Fermentation With *Hirsutella sinensis* and Multi‐Strains Synergistic Fermentation.” Innovative Food Science & Emerging Technologies 103: 104059. 10.1016/j.ifset.2025.104059.

[fsn372445-bib-0012] Guo, X. , C.‐T. Ho , W. Schwab , and X. Wan . 2021. “Effect of the Roasting Degree on Flavor Quality of Large‐Leaf Yellow Tea.” Food Chemistry 347: 129016. 10.1016/j.foodchem.2021.129016. 33486364

[fsn372445-bib-0013] Hu, W. , G. Wang , S. Lin , et al. 2022. “Digital Evaluation of Aroma Intensity and Odor Characteristics of Tea With Different Types—Based on OAV‐Splitting Method.” Food 11, no. 15: 2204. 10.3390/foods11152204. PMC9329961 35892790

[fsn372445-bib-0014] Hua, J. , W. Ouyang , X. Zhu , et al. 2024. “Objective Quantification Technique and Widely Targeted Metabolomic Reveal the Effect of Drying Temperature on Sensory Attributes and Related Non‐Volatile Metabolites of Black Tea.” Food Chemistry 439: 138154. 10.1016/j.foodchem.2023.138154. 38071844

[fsn372445-bib-0015] Huang, J. , J. Zhang , Z. Chen , et al. 2024. “Sensory‐Directed Flavor Analysis of Jinggu White Tea: Exploring the Formation Mechanisms of Sweet and Fruity Aromas.” Food Chemistry: X 24: 102026. 10.1016/j.fochx.2024.102026. PMC11626070 39655215

[fsn372445-bib-0016] Huang, W. , S. Fang , J. Wang , et al. 2022. “Sensomics Analysis of the Effect of the Withering Method on the Aroma Components of Keemun Black Tea.” Food Chemistry 395: 133549. 10.1016/j.foodchem.2022.133549. 35777211

[fsn372445-bib-0017] Jiang, H. , F. Yu , L. Qin , et al. 2019. “Dynamic Change in Amino Acids, Catechins, Alkaloids, and Gallic Acid in Six Types of Tea Processed From the Same Batch of Fresh Tea ( *Camellia sinensis* L.) Leaves.” Journal of Food Composition and Analysis 77: 28–38. 10.1016/j.jfca.2019.01.005.

[fsn372445-bib-0018] Li, H. , Z.‐W. Liu , Z.‐J. Wu , Y.‐X. Wang , R.‐M. Teng , and J. Zhuang . 2018. “Differentially Expressed Protein and Gene Analysis Revealed the Effects of Temperature on Changes in Ascorbic Acid Metabolism in Harvested Tea Leaves.” Horticulture Research 5, no. 1: 65. 10.1038/s41438-018-0070-x. PMC6165846 30302261

[fsn372445-bib-0019] Li, W. , Z. Zhang , R. Chen , et al. 2025. “Metabolomics‐Based Analysis of the Effects of Differences in Soluble Sugars on the Sweetness Quality of Six Major Tea Types in China.” Food & Function 16, no. 9: 3707–3720. 10.1039/D5FO00232J. 40259751

[fsn372445-bib-0020] Liang, S. , Y. Gao , D. Granato , et al. 2024. “Pruned Tea Biomass Plays a Significant Role in Functional Food Production: A Review on Characterization and Comprehensive Utilization of Abandon‐Plucked Fresh Tea Leaves.” Comprehensive Reviews in Food Science and Food Safety 23, no. 4: e13406. 10.1111/1541-4337.13406. 39030800

[fsn372445-bib-0021] Liang, S. , F. Wang , D. Granato , et al. 2023. “Effect of β‐Glucosidase on the Aroma of Liquid‐Fermented Black Tea Juice as an Ingredient for Tea‐Based Beverages.” Food Chemistry 402: 134201. 10.1016/j.foodchem.2022.134201. 36122474

[fsn372445-bib-0022] Lin, F. , X. Wei , H. Liu , et al. 2021. “State‐Of‐The‐Art Review of Dark Tea: From Chemistry to Health Benefits.” Trends in Food Science & Technology 109: 126–138. 10.1016/j.tifs.2021.01.030.

[fsn372445-bib-0023] Lin, H. , S. Ye , J. Feng , et al. 2025. “Impact of Compression Methods on Flavor Profile of White Tea: Integrated Analysis of Appearance, Aroma, and Taste.” Food Chemistry: X 28: 102510. 10.1016/j.fochx.2025.102510. PMC12139221 40475816

[fsn372445-bib-0024] Lin, J. , Y. Wang , L. Chen , Y. Yang , Z. Tu , and Y. Ye . 2022. “Effect of the Presence of Stem on Quality of Oolong Tea.” Food 11, no. 21: 3439. 10.3390/foods11213439. PMC9657438 36360052

[fsn372445-bib-0025] Liu, C. , J. Li , H. Li , et al. 2023. “Differences in the Quality of Black Tea ( *Camellia sinensis* Var. Yinghong No. 9) in Different Seasons and the Underlying Factors.” Food Chemistry: X 20: 100998. 10.1016/j.fochx.2023.100998. PMC10739754 38144863

[fsn372445-bib-0026] Liu, F. , H. Zhu , C. Li , et al. 2025. “A Combined Drying Process Involving Hot Air and Roasting for Improving Summer Congou Black Tea Quality.” Food Research International 201: 115584. 10.1016/j.foodres.2024.115584. 39849739

[fsn372445-bib-0027] Liu, H. , Y. Xu , J. Wen , et al. 2021. “A Comparative Study of Aromatic Characterization of Yingde Black Tea Infusions in Different Steeping Temperatures.” LWT 143: 110860. 10.1016/j.lwt.2021.110860.

[fsn372445-bib-0028] Luo, J. , S. Liao , F. Ding , et al. 2025. “Effects of Various Drying Parameters on the Volatile and Non‐Volatile Compositions of ‘Qiancha 1’ White Tea.” Food 14, no. 21: 3787. 10.3390/foods14213787. PMC12610232 41227757

[fsn372445-bib-0029] Ma, L. , Y. Sun , X. Wang , et al. 2023. “The Characteristic of the Key Aroma‐Active Components in White Tea Using gc‐tof ‐ ms and gc‐ Olfactometry Combined With Sensory‐Directed Flavor Analysis.” Journal of the Science of Food and Agriculture 103, no. 14: 7136–7152. 10.1002/jsfa.12798. 37337850

[fsn372445-bib-0030] Ma, Q. , M. Qin , L. Song , et al. 2022. “Molecular Link in Flavonoid and Amino Acid Biosynthesis Contributes to the Flavor of Changqing Tea in Different Seasons.” Food 11, no. 15: 2289. 10.3390/foods11152289. PMC9368528 35954056

[fsn372445-bib-0031] Murray, A. F. , A. J. Moore , and J. P. Munafo . 2020. “Key Odorants From the American Matsutake, *Tricholoma magnivelare* .” Journal of Agricultural and Food Chemistry 68, no. 36: 9768–9775. 10.1021/acs.jafc.0c03372. 32840362

[fsn372445-bib-0032] Qin, D. , Q. Wang , H. Li , et al. 2020. “Identification of Key Metabolites Based on Non‐Targeted Metabolomics and Chemometrics Analyses Provides Insights Into Bitterness in Kucha [Camellia Kucha (Chang et Wang) Chang].” Food Research International 138: 109789. 10.1016/j.foodres.2020.109789. 33288175

[fsn372445-bib-0033] Ren, Z.‐W. , H.‐J. Pan , C. Hu , et al. 2024. “Rolling Forms the Diversities of Small Molecular Nonvolatile Metabolite Profile and Consequently Shapes the Bacterial Community Structure for Keemun Black Tea.” Food Research International 181: 114094. 10.1016/j.foodres.2024.114094. 38448096

[fsn372445-bib-0034] Shen, J. , Q. Liu , J. Ning , and L. Li . 2025. “Elucidating the Taste Profile in Black Tea Through Molecular Docking and Molecular Sensory Science.” Food Chemistry 491: 145104. 10.1016/j.foodchem.2025.145104. 40543126

[fsn372445-bib-0035] Shi, J. , W. Wu , Y. Zhang , et al. 2023. “Comprehensive Analysis of Carotenoids Constituents in Purple‐Coloured Leaves and Carotenoid‐Derived Aroma Differences After Processing Into Green, Black, and White Tea.” LWT 173: 114286. 10.1016/j.lwt.2022.114286.

[fsn372445-bib-0036] Shi, Y. , M. Wang , Z. Dong , et al. 2021. “Volatile Components and Key Odorants of Chinese Yellow Tea ( *Camellia sinensis* ).” LWT 146: 111512. 10.1016/j.lwt.2021.111512.

[fsn372445-bib-0037] Song, L. , Q. Zhao , X. Shao , et al. 2025. “Identification of Characteristic Flavor Compounds and Quality Evaluation of *Butyriboletus roseoflavus* From Different Regions in Yunnan.” Food 14, no. 10: 1676. 10.3390/foods14101676. PMC12111782 40428457

[fsn372445-bib-0038] Su, D. , J.‐J. He , Y.‐Z. Zhou , Y.‐L. Li , and H.‐J. Zhou . 2022. “Aroma Effects of Key Volatile Compounds in Keemun Black Tea at Different Grades: HS‐SPME‐GC‐MS, Sensory Evaluation, and Chemometrics.” Food Chemistry 373: 131587. 10.1016/j.foodchem.2021.131587. 34838407

[fsn372445-bib-0039] Sun, Z. , Y. Lin , H. Yang , R. Zhao , J. Zhu , and F. Wang . 2024. “Characterization of Honey‐Like Characteristic Aroma Compounds in Zunyi Black Tea and Their Molecular Mechanisms of Interaction With Olfactory Receptors Using Molecular Docking.” LWT 191: 115640. 10.1016/j.lwt.2023.115640.

[fsn372445-bib-0040] Tu, Z. , S. Li , A. Xu , et al. 2025. “Improvement of Summer Green Tea Quality Through an Integrated Shaking and Piling Process.” Food 14, no. 7: 1284. 10.3390/foods14071284. PMC11989215 40238596

[fsn372445-bib-0041] van Gemert, L. J. 2011. Compilations of Flavour Threshold Values in Water and Other Media (Sendond Enlarged and Revised Edition). Oliemans Punter & Partners BV.

[fsn372445-bib-0042] Wang, H. , X. Cao , Z. Yuan , and G. Guo . 2021. “Untargeted Metabolomics Coupled With Chemometrics Approach for Xinyang Maojian Green Tea With Cultivar, Elevation and Processing Variations.” Food Chemistry 352: 129359. 10.1016/j.foodchem.2021.129359. 33735748

[fsn372445-bib-0043] Wang, T. , N. Bo , Y. Guan , et al. 2025. “An Integrated Flavoromics and Chemometric Analysis of the Characteristic Flavor, Chemical Basis and Flavor Wheel of Ancient Plant Ripened Pu‐Erh Tea.” Food Chemistry: X 26: 102278. 10.1016/j.fochx.2025.102278. PMC11880732 40046997

[fsn372445-bib-0045] Wei, Y. , X. Yin , H. Wu , et al. 2022. “Improving the Flavor of Summer Green Tea ( *Camellia sinensis* L.) Using the Yellowing Process.” Food Chemistry 388: 132982. 10.1016/j.foodchem.2022.132982. 35447593

[fsn372445-bib-0046] Wen, L. , L. Sun , R. Chen , et al. 2023. “Metabolome and Microbiome Analysis to Study the Flavor of Summer Black Tea Improved by Stuck Fermentation.” Food 12, no. 18: 3414. 10.3390/foods12183414. PMC10527649 37761123

[fsn372445-bib-0047] Wen, S. , L. Sun , S. Zhang , et al. 2023. “The Formation Mechanism of Aroma Quality of Green and Yellow Teas Based on GC‐MS/MS Metabolomics.” Food Research International 172: 113137. 10.1016/j.foodres.2023.113137. 37689901

[fsn372445-bib-0048] Wu, H. , Y. Chen , W. Feng , et al. 2022. “Effects of Three Different Withering Treatments on the Aroma of White Tea.” Food 11, no. 16: 2502. 10.3390/foods11162502. PMC9407123 36010502

[fsn372445-bib-0049] Wu, L. , X. Huang , S. Liu , et al. 2020. “Understanding the Formation Mechanism of Oolong Tea Characteristic Non‐Volatile Chemical Constitutes During Manufacturing Processes by Using Integrated Widely‐Targeted Metabolome and DIA Proteome Analysis.” Food Chemistry 310: 125941. 10.1016/j.foodchem.2019.125941. 31835227

[fsn372445-bib-0050] Xu, J. , M. Wang , J. Zhao , Y.‐H. Wang , Q. Tang , and I. A. Khan . 2018. “Yellow Tea ( *Camellia sinensis* L.), a Promising Chinese Tea: Processing, Chemical Constituents and Health Benefits.” Food Research International 107: 567–577. 10.1016/j.foodres.2018.01.063. 29580521

[fsn372445-bib-0051] Yang, T. , T. Zhang , S. Fang , et al. 2024. “Effect of Spreading Time on the Taste Quality of Steamed Green Tea Based on E‐Tongue Evaluation and Chemometric Statistical Analysis.” Journal of Food Measurement and Characterization 18, no. 5: 3176–3188. 10.1007/s11694-024-02394-0.

[fsn372445-bib-0052] Yang, Z. , S. Baldermann , and N. Watanabe . 2013. “Recent Studies of the Volatile Compounds in Tea.” Food Research International 53, no. 2: 585–599. 10.1016/j.foodres.2013.02.011.

[fsn372445-bib-0053] Yu, Y. , X. Feng , I. Blank , et al. 2025. “A Review of Umami Taste of Tea: Substances, Perception Mechanism, and Physiological Measurement Prospects.” Trends in Food Science & Technology 162: 105082. 10.1016/j.tifs.2025.105082.

[fsn372445-bib-0054] Zhang, L. , Q.‐Q. Cao , D. Granato , Y.‐Q. Xu , and C.‐T. Ho . 2020. “Association Between Chemistry and Taste of Tea: A Review.” Trends in Food Science & Technology 101: 139–149. 10.1016/j.tifs.2020.05.015.

[fsn372445-bib-0055] Zhang, X. , X. Du , Y. Li , et al. 2022. “Are Organic Acids Really Related to the Sour Taste Difference Between Chinese Black Tea and Green Tea?” Food Science & Nutrition 10, no. 6: 2071–2081. 10.1002/fsn3.2823. PMC9179145 35702304

[fsn372445-bib-0056] Zhou, F. , M. Zhu , J. Tang , et al. 2022. “Six Types of Tea Extracts Attenuated High‐Fat Diet‐Induced Metabolic Syndrome via Modulating Gut Microbiota in Rats.” Food Research International 161: 111788. 10.1016/j.foodres.2022.111788. 36192879

[fsn372445-bib-0058] Zhu, W. , Y. Jiang , Y. Zhu , et al. 2025. “Quality Assessment of Jasmine Tea Combining Computer Vision and Color Difference Analysis.” Chemistry & Biodiversity 22, no. 11: e00905. 10.1002/cbdv.202500905. 40674626

[fsn372445-bib-0059] Zhu, Y. , H.‐P. Lv , C.‐Y. Shao , et al. 2018. “Identification of Key Odorants Responsible for Chestnut‐Like Aroma Quality of Green Teas.” Food Research International 108: 74–82. 10.1016/j.foodres.2018.03.026. 29735103

[fsn372445-bib-0060] Zou, G. , Y. Xiao , M. Wang , and H. Zhang . 2018. “Detection of Bitterness and Astringency of Green Tea With Different Taste by Electronic Nose and Tongue.” PLoS One 13, no. 12: e0206517. 10.1371/journal.pone.0206517. PMC6312292 30596674

[fsn372445-bib-0044] 王, X. , X. 李 , and B. 刘 . 2021. “ *冠囊散囊菌* 介导的夏秋茶纯发酵与混合发酵 化学成分比较.” LWT‐食品科学与技术 143: 111132. 10.1016/j.lwt.2021.111132.

